# Preclinical development of a first-in-class vaccine encoding HER2, Brachyury and CD40L for antibody enhanced tumor eradication

**DOI:** 10.1038/s41598-023-32060-2

**Published:** 2023-03-30

**Authors:** Maria Hinterberger, Kathrin Endt, Barbara Bathke, Matthias Habjan, Alexander Heiseke, Marc Schweneker, Julia Von Rohrscheidt, Cigdem Atay, Paul Chaplin, Markus Kalla, Jürgen Hausmann, Carolin Schmittwolf, Henning Lauterbach, Ariane Volkmann, Hubertus Hochrein, José Medina-Echeverz

**Affiliations:** 1 Bavarian Nordic GmbH, Fraunhoferstr.13, 82152 Planegg, Germany; 2 Present Address: Origenis GmbH, Am Klopferspitz 19A, 82152 Planegg, Germany; 3 Present Address: GlaxoSmithKline GmbH, Prinzregentenpl. 9, 81675 Munich, Germany; 4 Present Address: Hookipa Pharma Inc, 350 Fifth Avenue, Room/Suite 7240, New York City, NY USA; 5 Present Address: Affimed, Im Neuenheimer Feld 582, 69120 Heidelberg, Germany

**Keywords:** Cancer, Immunology

## Abstract

The induction of antiviral innate immunity by systemic immunization with live virus can be employed to positively impact the response to therapeutic vaccination. We previously demonstrated that systemic immunization with a non-replicating MVA encoding CD40 ligand (CD40L) enhances innate immune cell activation and function, and triggers potent antitumor CD8^+^ T cell responses in different murine tumor models. Antitumor efficacy was increased when combined with tumor targeting antibodies. Here we report the development of TAEK-VAC-HerBy (TVH), a first-in-class human tumor antibody enhanced killing (TAEK) vaccine based on the non-replicating MVA-BN viral vector. It encodes the membrane bound form of human CD40L, HER2 and the transcription factor Brachyury. TVH is designed for therapeutic use in HER2- or Brachyury-expressing cancer patients in combination with tumor targeting antibodies. To preclude possible oncogenic activities in infected cells and to prevent binding of vaccine-encoded HER2 by monoclonal antibodies trastuzumab and pertuzumab, genetic modifications of HER2 were introduced in the vaccine. Brachyury was genetically modified to prevent nuclear localization of the protein thereby inhibiting its transcriptional activity. CD40L encoded in TVH enhanced human leukocyte activation and cytokine secretion in vitro. Lastly, TVH intravenous administration to non-human primates was proven immunogenic and safe in a repeat-dose toxicity study. Nonclinical data presented here highlight TVH as a first-in-class immunotherapeutic vaccine platform currently under clinical investigation.

## Introduction

Therapeutic cancer vaccines are designed to generate de novo and expand pre-existing CD4^+^ and CD8^+^ T cell responses specific for tumor associated antigens (TAAs). Live viral vectors represent an excellent vaccine platform to deliver TAAs and stimulate the immune system. Viral infection, recognition and death of infected cells trigger the activation of antiviral defense mechanisms that modulate antitumor innate and adaptive immune responses.

Modified Vaccinia Ankara (MVA)-BN is a highly attenuated vaccinia strain, replication deficient in humans and other mammals, including immunocompromised mice and non-human primates (NHPs), and is approved as a third-generation smallpox and monkeypox vaccine in USA (JYNNEOS™), Canada (IMVAMUNE®) and the European Union (IMVANEX®)^1^. Furthermore, the recombinant MVA based Ebola vaccine MVABEA® (MVA-BN Filo) administered in combination with Janssen's Ebola vaccine ZABDENO® (Ad26.ZEBOV) was recently approved by the European Medicines Agency (EMA)^2^. MVA is currently being employed in clinical trials against infectious diseases^3^ and in patients with cancer^4^. Hence, MVA-BN’s excellent safety profile in humans^5,6^, the maintained capability to induce strong and durable cellular and humoral immune responses^1,7^, and the capacity to express numerous transgenes make it an attractive vaccine platform.

Human epidermal growth factor receptor 2 (HER2) amplification and overexpression play a key role in many epithelial tumor types including breast and gastric cancer^8,9^. These tumors have a distinctive molecular signature which is associated with aggressive biological behavior and poor clinical outcome. HER2 is considered a driver oncogene promoting cancer cell growth. Accordingly, inhibition of the HER2 signaling pathway translates into clinical benefit. In addition to the fact that HER2 is a target for cell signaling inhibition, HER2 is also a potential target for immunotherapy^10^ in the context of combination therapies^11^. However, a substantial fraction of patients is primarily resistant or will become resistant to HER2 targeting drugs and ultimately will end up dying of cancer^10,12^.

Brachyury is a member of the T-box family of transcription factors, characterized by a highly conserved DNA-binding domain designated as T domain^13,14^. Brachyury is a driver of epithelial-to-mesenchymal transition (EMT). EMT is a reversible process during which cells switch from a polarized, epithelial phenotype into a highly motile, mesenchymal phenotype^15^. It has been demonstrated that overexpression of Brachyury in human carcinoma cell lines is able to drive a switch from an epithelial to a mesenchymal-like phenotype^16^. In addition, Brachyury is highly overexpressed in the rare bone sarcoma chordoma^17^. Together with differential Brachyury expression mostly in tumor tissues^18^, these findings make Brachyury an attractive target for cancer vaccine development. MVA vector-based vaccines targeting Brachyury are currently under clinical evaluation^19,20^.

The TNF receptor family member CD40 is considered a costimulatory master-switch of dendritic cells (DCs). CD40-CD40 ligand (CD40L)-based interaction of DCs with activated CD4^+^ T cells `license´ DCs to prime CD8^+^ T cell responses^21^. Our previous work showed that systemic immunization with a non-replicating MVA encoding CD40L (MVA-CD40L) enhanced antitumor immune responses in different tumor models and mouse strains^22^. In addition, immunization with MVA-CD40L modulated the production of proinflammatory cytokines and expanded and activated natural killer (NK) cells systemically. Indeed, therapeutic combination of MVA-CD40L with antibodies targeting TAAs significantly improved antitumor responses against established, aggressive carcinomas, relying on a NK cell- and Fc receptor-dependent mechanism.

In this study we describe the development of a novel human tumor antibody enhanced killing (TAEK) vaccine (VAC) encoding the membrane bound form of human CD40L, HER2 (Her) and the human transcription factor Brachyury (By), (TAEK-VAC-HerBy, TVH). It is designed for intravenous administration in patients with advanced HER2-or Brachyury-expressing cancers and to be combined with HER2-targeting therapeutic antibodies.

TVH carries genetic modifications of HER2 to prevent possible oncogenic activities in infected cells. Additionally, vaccine encoded HER2 cannot be bound by the monoclonal antibodies trastuzumab and pertuzumab due to modifications in the respective binding epitopes. Brachyury was genetically modified to prevent translocation of the protein into the nucleus and thereby inhibits its transcriptional activity. TVH infection in vitro led to potent human leukocyte and DC activation and cytokine secretion. In addition, TVH administered intravenously to NHPs was immunogenic and showed a favorable safety profile in a repeat-dose toxicity study. Nonclinical data presented here highlight TVH as a first-in-class immunotherapeutic vaccine which currently undergoes clinical testing (NCT04246671).

## Results

### Generation of TAEK-VAC-HerBy

**Figure 1:**
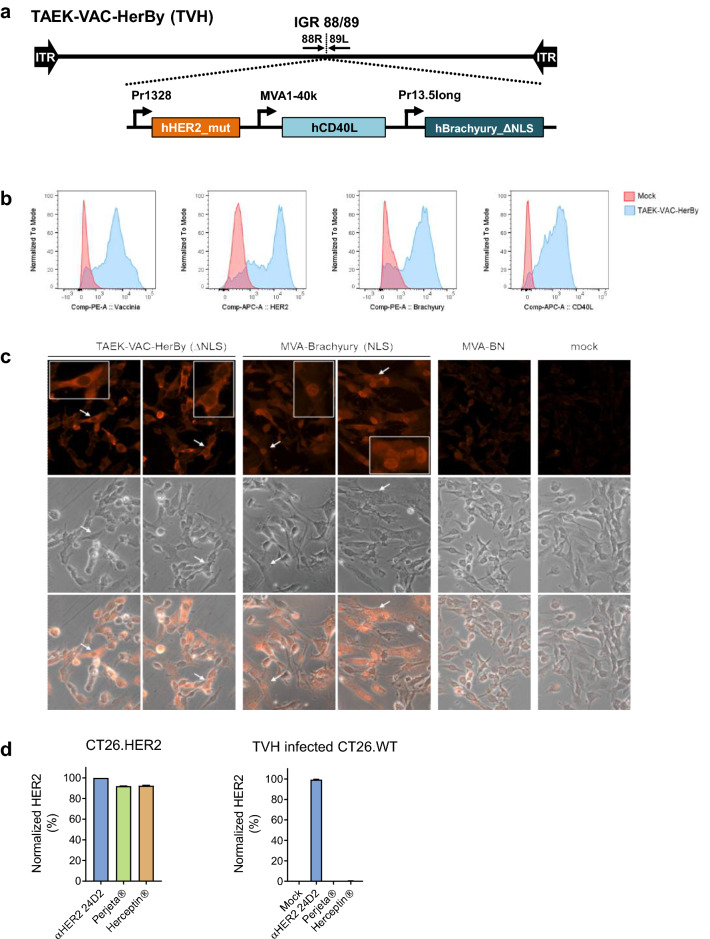
Characterization of transgenes contained in TAEK-VAC-HerBy. **(a)** Schematic map of TAEK-VAC-HerBy showing the transgenes in the Intergenic Region (IGR) 88/89 of the MVA-BN genome. Expression of transgenes is controlled by different poxvirus promoters; **(b)** Transgene expression detected by flow cytometry. Briefly, HeLa cells were infected overnight either with mock or with TVH at a MOI of 4. Expression of vaccinia, HER2, Brachyury and CD40L (blue histogram) against mock control (red histogram) is shown; **(c)** Analysis of subcellular localization of Brachyury-ΔNLS and Brachyury-NLS by immunofluorescence staining. A31 cells were either mock infected or infected at a MOI of 2 with MVA-BN, MVA-Brachyury or TVH. At 17 h post infection, cells were fixed, permeabilized, and stained with anti-Brachyury antibody followed by PE-conjugated secondary antibody. Upper panel represents Brachyury staining, followed by clear field (middle panel) and overlay (lower panel). To better show subcellular localization of Brachyury-ΔNLS or Brachyury-NLS, inlays display higher magnification of representative cells indicated by arrow; **(d)** Differential binding of HER2 antibodies to mutated HER2 present in TVH. Briefly, CT26.WT cells were infected overnight with TVH at a MOI of 5. Then, infected CT26.WT cells as well as control CT26.HER2 cells were stained with different HER2 antibody clones, namely HER2 24D2, Herceptin® or Perjeta®. Data show HER2 expression detected by Herceptin® and Perjeta® normalized to HER2 24D2-mediated detection. Data shown in B-D are representative of several independent experiments.

TVH is a recombinant immunotherapy vaccine based on the non-replicating MVA-BN viral vector, which itself is licensed as smallpox and monkeypox vaccine under the trade name JYNNEOS®. It encodes the human HER2, the human transcription factor Brachyury and the membrane bound form of human CD40L in the Intergenic Region (IGR) 88/89 of the MVA-BN genome (Fig. 1a). Expression of the transgenes is controlled by different poxvirus promoters. HER2 was genetically modified to preclude possible oncogenic activities in TVH-infected cells and to prevent binding of monoclonal antibodies trastuzumab and pertuzumab to TVH-encoded HER2. Brachyury was genetically modified to prevent translocation of the protein into the nucleus and thereby inhibit its transcriptional activity. All encoded transgenes were detected in TVH-infected HeLa cells (Fig. 1b).

Brachyury is required in the early determination and differentiation of mesoderm. Mutations in this transcription factor have been shown to result in vertebral malformations. In addition, dysregulation of Brachyury may be involved in the formation of chordomas, malignant tumors in the spine. Moreover, it is reported to play a key role in the metastasis and progression of tumors by driving endothelial-mesenchymal transition of tumor cells^23^. To avoid potential issues caused by the function of Brachyury as a transcription factor its nuclear localization signal was deleted in TVH to retain it in the cytoplasm^24^. Moreover, Brachyury antigen cross-presentation might conceivably be more efficient with cytoplasmic-expressed Brachyury when compared to Brachyury that is localized in the nucleus. To analyze subcellular localization of Brachyury, BALB/3T3 clone A31 cells were infected with TVH, lacking the potential nuclear localization signal (NLS) of Brachyury (ΔNLS), or with a recombinant MVA encoding Brachyury still containing its NLS (MVA-Brachyury) for comparison. Subcellular distribution of Brachyury-ΔNLS encoded by TVH was confined to the cytoplasm, as the nucleus was devoid of staining (Fig. 1c). In contrast, in cells infected with MVA-Brachyury, Brachyury-specific staining diffused throughout the cells with the strongest staining in the nucleus, indicating that Brachyury was present both in the nucleus and cytoplasm. Specificity of staining was shown with cells that had been infected with MVA-BN or with mock infected cells.

Two humanized monoclonal antibodies targeting HER2, trastuzumab and pertuzumab, have been developed and approved for treatment of HER2-overexpressing malignancies^25,26^. These antibodies bind to different sites in the extracellular domain of HER2. Trastuzumab binding results in signal transduction blockade and prevention of HER2 cleavage^27,28^. In contrast, pertuzumab sterically blocks HER2 dimerization with other EGF receptors and blocks ligand-activated signaling^29^. Both antibodies in combination provide a dual blockade of HER2-driven signaling pathways and in addition can mediate antibody-dependent cell cytotoxicity (ADCC). The rationale behind TVH involves administration of MVA with trastuzumab and/or pertuzumab. Consequently, binding of these antibodies to the MVA-encoded HER2 must be prevented. Likewise, any adverse effects which might result from HER2 overexpression by MVA in targeted cells need to be avoided.

Therefore, residues in both trastuzumab (Herceptin®) and pertuzumab (Perjeta®) binding sites were mutated (Supplementary Fig. 1). Interaction of trastuzumab with HER2 requires 3 loops in the juxtamembrane region of HER2 formed by amino acids 578–582 (loop1), 591–594 (loop2), as well as 614–624 (loop3). Based on structure analysis and relevant literature search the key residues E580, F595 as well as K615 were mutated to alanine in TVH^30,31^, leading to a loss of binding of vaccine-encoded HER2 by trastuzumab (Fig. 1d, right). Pertuzumab binds close to a loop in the dimerization domain (domain II) of HER2^29,32^. Initially, we mutated the two pertuzumab binding sites L317 and H318 to alanine, resulting in only partial loss of pertuzumab binding to mutated HER2 (data not shown). Hence, a total of eight amino residues were mutated to alanine: H267, F279, V308, S310, L317, H318, K333, P337 which completely abolished binding of pertuzumab to TVH-expressed HER2 (Fig. 1d, right). In contrast to anti-HER2 antibody clone 24D2, both trastuzumab (Herceptin®) and pertuzumab (Perjeta®) did not bind to the modified HER2 expressed by TVH (Fig. 1d, right), whereas unmodified HER2 was readily detected using either anti-HER2 antibody clone 4D2, trastuzumab or pertuzumab in cells constitutively expressing natural unmodified HER2 (CT26.HER2) (Fig. 1d, left). To avoid any potential adverse effects upon HER2 overexpression by TVH, (i) the extracellular dimerization domain was mutated, (ii) the tyrosine kinase activity was inactivated, and (iii) potential phosphorylation sites were eliminated^33,34^.

### TVH activates human immune cells

**Figure 2:**
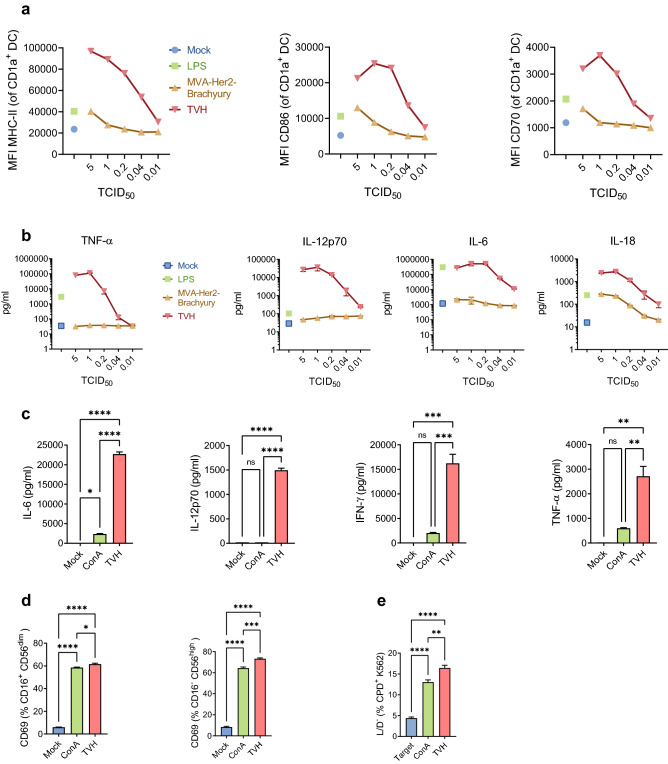
TVH activates innate immune cells in vitro. **(a, b)** Activation and cytokine production of dendritic cells upon TVH infection. Monocyte-derived DCs (mo-DC) were generated after enrichment of CD14^+^ monocytes from human PBMCs and cultured for 7 days in the presence of GM-CSF and IL-4 as explained in *Materials and Methods*. Mo-DCs were infected overnight either with MVA-BN or TVH at different MOIs. LPS and mock infections were used as positive and negative controls, respectively. Supernatants were then collected, and cells were stained for flow cytometry analysis. **(a)** Mean Fluorescence Intensity (MFI) of MHC class II (MHC-II), CD86 and CD70 gated on CD1a^+^ DCs; **(b)** TNF-α, IL-12p70, IL-6 and IL-18 quantification in supernatants. A representative donor out of four tested is shown; **(c-e)** Cryopreserved PBMCs from the same donor were infected with TVH at a MOI of 0.5 or stimulated with ConA as a positive control. After overnight incubation, supernatants were collected, and cells were either stained for flow cytometry analysis or NK cells were magnetically sorted. Sorted NK cells were incubated with Cell Proliferation Dye (CPD) labelled K562 target cells in a 1:1 ratio (effector:target). **(c)** Cytokine secretion profile showing IL-6, IL-12p70, IFN-γ and TNF-α; **(d)** Frequency of CD69 positive cells on CD16^+^CD56^low^ and CD16^-^CD56^high^ NK cells detected by flow cytometry, indicating TVH-induced activity and cytotoxicity of NK cells. **(e)** Percentage of dead CPD K562 target cells after being incubated with sorted NK cells. Data are expressed as Mean ± SEM and are representative of two independent experiments. One-way ANOVA was performed on figures c-e. ns, non-significant; *, *p* < 0.05; **p < 0.01; ***p < 0.005; ****p < 0.001.

We have previously reported that a recombinant MVA expressing CD40L induced murine DC activation in vitro^21^. Hence, we next tested whether human CD40L encoded in TVH would induce activation and maturation of human DCs. Transduction of human monocyte-derived DCs from healthy donors using TVH resulted in surface expression of HER2 and CD40L (Supplementary Fig. 2). Increased mean fluorescence intensity (MFI) of CD86, MHC-II and CD70 was observed upon overnight incubation of infected DCs with TVH and depending on TCID_50_ used for infection (Fig. 2a). This effect was not seen when using a recombinant MVA expressing HER2 and Brachyury but no CD40L. Cytokine analysis of supernatants revealed that TVH but not MVA encoding only HER2 and Brachyury induced expression of IL-12p70, IL-6 and TNF-α and significantly boosted IL-18 secretion (Fig. 2b). Surface marker and cytokine expression peaked at a TCID_50_ of 1, indicating cytopathic and/or inhibitory effects of MVA on DCs at higher doses.

Next, we addressed whether TVH infection of peripheral blood mononuclear cells (PBMCs) would result in immune cell activation. PBMCs were incubated with TVH or ConA, which was used as a positive control. Supernatants were analyzed in terms of cytokine secretion profile. TVH infection induced IL-12p70, IFN-γ, IL-6 and TNF-α and to a higher level than in response to ConA (Fig. 2c). We have previously demonstrated that CD40L encoded by MVA enhanced murine NK cell activation in vivo by means of increased CD69 expression^22^. Similarly, we found increases in CD69^+^ frequencies of CD16^-^CD56^high^ and CD16^+^CD56^dim^ human NK cells upon PBMC infection with TVH (Fig. 2d). Next, we assessed TVH-induced NK cell lytic function against K562 cells, a human leukemia cell line that lacks MHC-I and is classically used to test NK cell cytotoxicity. Incubation of K562 cells with sorted NK cells from PBMCs either infected with TVH or activated with ConA resulted in a significant induction of tumor cell death (Fig. 2e). In summary, TVH infection activates human immune cells, driving both DC and NK cell activation, and enhancing NK lytic activity.

### TVH infection results in vaccine encoded peptide loading onto HLA molecules

**Figure 3:**
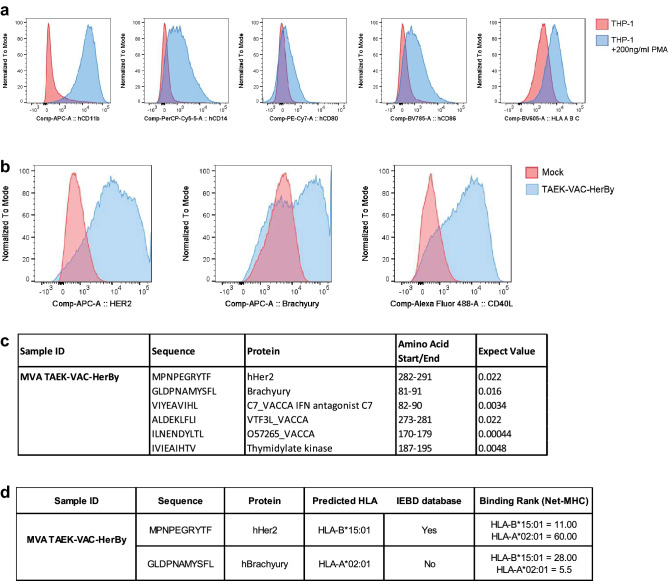
Brachyury and HER2 expressed in TVH are loaded onto HLA class I molecules.** (a, b)** TVH infection of THP-1-derived macrophages. THP-1 cells are cultured in the presence of 200 ng/ml PMA for 72 h. Then, cells are washed and rested in complete medium for additional 48 h until the start of the experiment. **(a)** Phenotype of THP-1-derived macrophages compared to undifferentiated THP-1 cells. Expression of hCD16, hCD14, hCD80, hCD86 and HLA-ABC is detected by flow cytometry. **(b)** Expression of HER2, Brachyury and CD40L on THP-1-derived macrophages. THP-1-derived macrophages were infected with TVH at Inf.U 4/cell. After overnight incubation, expression of HER2, Brachyury and CD40L was analysed by flow cytometry. **(c-d)** Analysis of peptides loaded onto HLA-ABC by mass spectrometry, and brief results of ProImmune ProPresent® MAPPS Antigen Presentation assay. Infected THP-1-derived macrophages are lysed, and HLA-peptide molecules recovered in an immune affinity step. Peptides are eluted from the HLA molecules and analysed by LC–MS/MS. Identified sequences are subjected to rigorous analysis to identify true positive peptides with high confidence. **(c)** Summary table of peptides detected by the ProPresent® Assay in TVH-infected macrophages. The likelihood of peptides to be real identities is described by their Expect Value. Peptides with an Expect Value ≤ 0.05 are indicative of identity; **(d)** Summary table for the features of TAA-derived peptides MPNPEGRYTF and GLDPNAMYSFL, showing predicted HLA-binding calculated using SYFPEITHI, whether they are listed in the IEDB database, and their binding rank to HLA-B*15:01 and HLA-A*02:01 defined by Net-MHC4.0.

The effective processing of vaccine-encoded antigens and loading on HLA molecules within antigen presenting cell (APC) as well as the surface presentation of immunogenic HLA/peptide complexes are key steps in the generation of adaptive immune responses by vaccines. To test whether TVH-derived antigens are presented on APC upon infection, we performed the ProPresent® antigen presentation assay with TVH infected human macrophages. We first differentiated the human monocytic cell line THP-1 into macrophages using Phorbol-12-Myristate-13-Acetate (PMA). As control, cellular markers of differentiation were analyzed (Fig. 3a). PMA-derived THP-1 macrophages were then infected with TVH and analyzed for transgene expression (Fig. 3b). To identify TVH-derived peptides, infected cells were lysed, panHLA Class 1 peptide complexes were immunoprecipitated, peptides were eluted und subjected to LC/MS mass spectrometry^35^.

Mass spectrometry analyses identified several MVA backbone-derived peptides that were loaded onto HLA Class 1 with an Expect Value of ≤ 0.05 (Fig. 3c)^36,37^. Interestingly, two tumor antigen-derived peptides were identified: the HER2-derived peptide MPNPEGRYTF^38^ and the Brachyury-derived peptide GLDPNAMYSFL (Fig. 3c). THP-1 cells were reported to express HLA-A*02:01:01G, HLA-B*15:01 and HLA-C*03^39^. Depending on the HLA anchor regions of a peptide, predictions can be made to which HLA it preferentially binds. Therefore, we performed an in silico analysis of both peptides to determine the potential HLA molecule it is associated to and the predicted binding strength of this interaction (Fig. 3d). HLA-B*15:01-bound peptides have a preferential usage of phenylalanine at the last position. Accordingly, the HER2 peptide MPNPEGRYTF is likely presented on HLA-B*15:01. In line with this, calculating the binding rank gives a lower value for HLA-B*15:01 than HLA-A*02:01:01, also indicating a better binding of this peptide to the former HLA (Fig. 3d). The Brachyury-derived peptide GLDPNAMYSFL has not been described before. The amino acid leucine at position 2 indicates a preferential binding to HLA-A*02:01. Even though this peptide is atypically long (11aa) for an HLA-A*02:01 binder, it has a strong predicted binding rate to this allele (Fig. 3d). Altogether, TVH infection resulted in the presentation of vaccine-derived tumor antigens by HLA molecules on human cells.

### Systemic administration of TVH to NHPs is proven safe

To interrogate the safety and immunogenicity profile of systemic TVH administration, we performed a repeated-dose toxicity study in male and female cynomolgus monkeys. Two different doses of TVH were tested by IV injection (Low = 1 × 10^9^ Inf.U; High = 6.75 × 10^9^ Inf.U) and Tris-buffered saline (TBS) was administered as control as summarized in Table 1. Assessment of toxicity indicated that intravenous TVH administration caused several clinical observations, as well as changes in body temperature, hematology, coagulation parameters and in clinical chemistry. All these parameters are summarized in Supplementary Table 1. For most of the findings in clinical pathology, it is assumed that these were induced by the pro-inflammatory pharmacological action of TVH. None of the clinical observations or findings in clinical or anatomic pathology were considered severe as many findings were reversible and did not impair the physical capacity of the animals or caused unexpected death. No findings in food consumption, assessment of feces consistency or electrocardiography were noted. In conclusion, the highest non-severely toxic dose (HNSTD) is considered the highest tested dose, 6.75 × 10^9^ Inf.U/animal, when administered intravenously three times in 3-week intervals.

**Table 1:** Study design: Toxicity of TAEK-VAC-HerBy, following three intravenous administrations of two different doses (Low, 1 × 10^9^ Inf.U; High, 6.75 × 10^9^ Inf.U) to cynomolgus monkeys (N = 5 male and 5 female/group) was assessed in 3-week intervals (vaccine administrations at Day 1, 22, 43). As control Tris-buffered saline (TBS) was used. An assessment of delayed onset toxicity and/or reversibility of toxicity was evaluated 3 days after the third administration and at the end of the recovery phase (Day 71).

Group	Dose	Dose Level (Inf.U/Animal)	Route	Animals/group		Necropsy on day	
				Males	Females	46	71^a^
A	Control	0	IV	5	5	3 M/3F	2 M/2F
B	Low	1 × 10^9^	IV	5	5	3 M/3F	2 M/2F
C	High	6.75 × 10^9^	IV	5	5	3 M/3F	2 M/2F

*IV* intravenous, *F* females, *M* males.

^a^Recovery group.

### TVH elicited innate immune responses in NHPs

**Figure 4:**
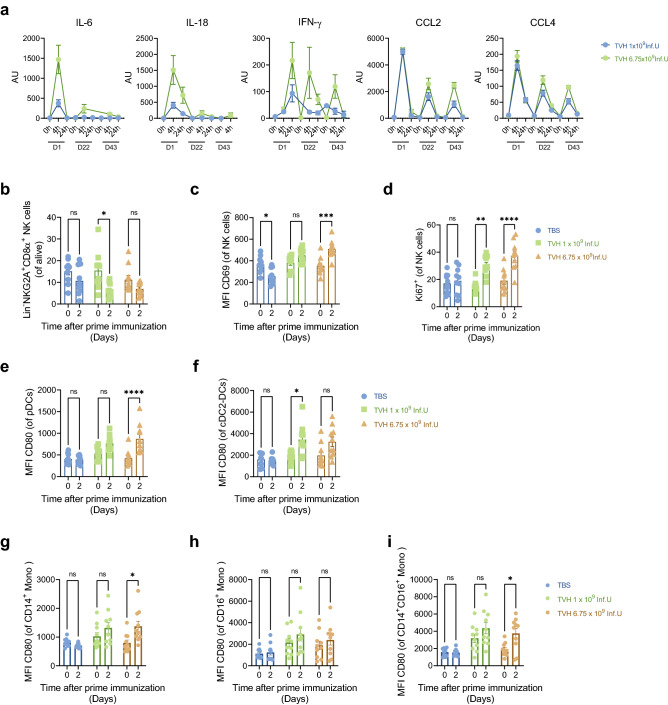
Intravenous injection of TVH into cynomolgus monkeys induces innate immune cell driven cytokine release and activation. TVH administration scheme was defined in Table 1. Briefly, 3 homologous intravenous injections of either TBS, 1 × 10^9^ Inf.U TVH or 6.75 × 10^9^ Inf.U TVH were performed on days 1, 22 and 43. Blood was taken prior to each immunization, 4 h and 24 h after each immunization for cytokine and chemokine analysis. For analysis of innate immune cell subset activation, PBMCs were taken on day 0 and day 2 after the first immunization. **(a)** Quantification of IL-6, IL-18, IFN-γ, CCL2 and CCL4 in sera at 0 (predose), 4 and 24 h after each immunization by Luminex is indicated as arbitrary units (AU). **(b)** Frequency of NK cells; **(c)** Mean Fluorescence Intensity (MFI) of CD69 on NK cells; **(d)** Percentage of Ki67^+^ NK cells; **(e)** MFI of CD80 on pDCs; (f) on cDC2-DCs, **(g)** CD14^+^ monocytes, **(h)** CD16^+^ monocytes, and **(i)** CD14^+^ CD16^+^ monocytes are shown for the times only after prime immunization. Data in figures b-i shown for individual animals and as Mean (depicted by columns) ± SEM. Two-way ANOVA was performe to evaluate statistically significant differences. ns, non-significant; *p < 0.05; **p < 0.01; ***p < 0.005; ****p < 0.001.

We next investigated whether systemic administration of TVH would induce innate immune responses in non-human primates (NHPs). We first analyzed the presence of cytokines in serum after each immunization. Interestingly, IFN-γ, IL-6, IL-18, CCL2 and CCL4 were detected between 4 and 24 h after TVH immunization (Fig. 4a), which is in line with our previous observations in mice^21,22^. For nearly all cytokines tested, the highest levels were obtained after the first administration and reduced, or absent levels were noted thereafter. Furthermore, the induction of all proinflammatory cytokines measured was dose-dependent.

We then analyzed the activation of NK cells and APCs among PBMCs which were isolated from blood taken before (0 h, Day 0) and after (48 h, Day 2) the first TVH administration. NK cells were identified as outlined in the gating strategy (Supplementary Fig. 3). Analysis of NK cells revealed a reduction in frequency even in TBS treated animals (Fig. 4b), indicating that this change might be rather procedural than test item related. Importantly, increased CD69 expression on NK cells was observed in both TVH doses administered (Fig. 4c). Increased frequencies of NK cells expressing Ki67 were observed after all TVH administrations (Fig. 4d). Therefore, systemic administration of TVH enhances NK cell activation in NHPs, in line with our previous reports^22,40^.

APC analysis was divided into DCs and monocytes by the gating strategy outlined in Supplementary Figs. 4 and 5, respectively. Briefly, three types of DCs were identified as Clec9A^+^ cDC1-DCs, CD1c^+^ cDC2-DCs and CD123^+^ pDCs as well as three types of monocytes: CD16^+^/CD14^-^ (CD16 SP) monocytes, CD16^+^/CD14^+^ (CD16/CD14 DP) monocytes and CD16^-^/CD14^+^ (CD14 SP) monocytes, similar to their human counterparts^41^. Activation of APCs was measured according to increased expression levels of the co-stimulatory markers CD40, CD80 and CD86. Of note, cDC1 were not detectable in any TVH dose 48 h after treatment. The remaining DC and monocyte subsets showed increased expression levels of the co-stimulatory markers CD80 (Fig. 4e–i), CD40 and CD86 (Supplementary Fig. 6). Altogether, our data indicate that intravenous administration of TVH induces innate immune cell activation in NHPs.

### Induction of humoral immunity by TVH

**Figure 5:**
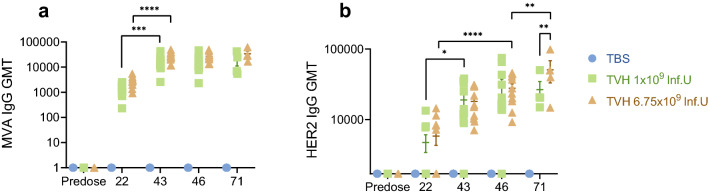
Induction of HER2- and MVA-specific IgG upon systemic immunization of cynomolgus monkeys using TVH. TVH administration scheme was defined in Table 1. Briefly, 3 homologous intravenous injections of either TBS, 1 × 10^9^ Inf.U TVH or 6.75 × 10^9^ Inf.U TVH were performed on days 1, 22 and 43. Blood was collected prior to the first immunization (predose: Day -12 and on days 22, 43, 46 and 71; day 71 only in Recovery Group; n = 4). Sera were analyzed using **(a)** MVA-specific IgG ELISA and **(b)** HER2-specific IgG ELISA. In this graph, IgG titers for individual animals are shown as well as GMTs (Geometric Mean Titers) depicted by horizonal bars +/-SEM. Two-way ANOVA was performed on figures a-b. ns, non-significant; *p < 0.05; **p < 0.01; ***p < 0.005; ****p < 0.001.

The aim of therapeutic vaccination is to modulate innate immune populations ultimately triggering the induction of humoral and cellular immune responses against vaccine-encoded antigens. Therefore, we investigated whether TVH would enhance vector backbone- and encoded tumor antigen-specific antibody titers in serum at day 22, 43, 46 and 71 compared to pre-dose titers. Administration of TVH induced MVA- and HER2-specific IgG responses that increased with repeated immunizations (Fig. 5). After a single administration none of the vaccinated groups showed complete seroconversion for HER2 specific antibodies. Overall, the second (day 22) and third (day 43) administration raised HER2-specific antibody levels significantly, irrespective of the dose administered (Fig. 5b). A significant difference between the low (group 2) and the high (group 3) dose could be observed when comparing bleeding date 43 and 71. A 1.1-fold and 1.7-fold higher HER2-specific antibody response in the high dose group compared to the low dose group was observed at these timepoints.

Taken together, intravenous TVH administration is safe and induces systemic innate immune activation as well as humoral immunity in NHPs.

## Discussion

In the present study, we characterized the immune components triggered by the cancer vaccine candidate TVH and described its toxicology and immunomodulatory profile in NHPs. We have previously reported the proof of concept and mechanisms of action underlying intravenous immunization using recombinant MVA encoding CD40L^22^. Based on these results, TVH was designed as a cancer vaccine to drive the systemic expansion and activation of innate immune cells including NK cells. TVH-induced innate immunity synergizes with activation of adaptive immune responses against vaccine-encoded TAAs and thus, triggers tumor eradication by cytotoxic CD8^+^ T cells.

TVH is a cancer vaccine aimed to treat HER2- and Brachyury-positive cancer patients in combination with HER2-targeting antibodies, such as trastuzumab and pertuzumab. The targeted mutations of both trastuzumab and pertuzumab binding sites prevent potential binding of these HER2 targeting antibodies to TVH infected cells^29–31^. This is a crucial feature as potential neutralization of virus or premature elimination of TVH-infected cells by the abovementioned therapeutic antibodies could abrogate or dampen TVH-induced innate and adaptive immune responses which would be detrimental for therapeutic efficacy. In addition, HER2 mutations in TVH reduce the risk of generating vaccine-driven HER2 specific antibodies that would compete with trastuzumab and/or pertuzumab binding sites. As the nature of TVH-induced antibodies is not known, there is a risk of generating antibodies that interfere with trastuzumab/pertuzumab binding but are not ADCC competent due to their isotype. Tumor targeting therapeutic antibodies have been selected for high specificity, affinity and ADCC competency among many different clones and are most likely superior in efficacy.

Epithelial-to-mesenchymal transition (EMT) seems to be involved in breast cancer progression and the ability of cells to switch between different phenotypes, known as epithelial-mesenchymal plasticity, is associated with resistance to targeted therapies^42–44^. In light of the potential involvement of EMT in breast cancer progression, the selection of a TAA associated with this process might be beneficial. Brachyury expression has been reported in different cancer types including breast and is thought to be a driver of the epithelial-to-mesenchymal transition^45^. It has been demonstrated that overexpression of brachyury in human carcinoma cell lines is able to drive a switch from an epithelial to a mesenchymal-like phenotype. Likewise, overexpression of brachyury in epithelial tumor cells also resulted in a concomitant increase in tumor cell migration and extracellular matrix invasion^23^. Brachyury expression has been reported in different cancer types such as lung, breast, ovarian, chordoma, prostate, colorectal and pancreatic adenocarcinoma^16,46,47^. Thus, targeting Brachyury can be a fruitful strategy to attack breast cancer cells that might have lost HER2 expression or dependency during metastasis and progression. Deletion of the nuclear localization sequence of the Brachyury protein led to localization of Brachyury exclusively in the cytoplasm of infected cells. By introducing this deletion, we not only sought to inhibit any potential Brachyury oncogenic signaling in the nucleus, but also to increase the likelihood of efficient antigen presentation to adaptive immune cells. Whereas nuclear and cytoplasmic location of antigens does not affect HLA-I presentation of antigens, efficient HLA-II presentation depends on cytosolic processing of antigen^48,49^. By targeting antigen localization to the cytosol, we expect to improve antigen presentation and loading onto HLA molecules. Indeed, we detected presentation of a Brachyury-derived peptide on HLA of infected human macrophages by mass spectrometric analyses of eluted peptides.

The main rationale of the cancer vaccine TVH is to combine it with tumor-targeting antibodies in order to deploy its full therapeutic capacity. Tumor-targeting antibodies have the ability to specifically recognize TAA and direct various components of the immune system against the tumor. ADCC is one of the central mechanisms in antibody-mediated cancer immunotherapy, involving NK cells^50^. Several FDA approved monoclonal antibodies mediate ADCC, including trastuzumab^51^ and pertuzumab, however, not all patients respond to ADCC-based therapies. Compounding this issue is the lack of NK cell infiltration into the tumor and inherently low efficacy observed with ADCC^52^. Thus, rational combination therapies that leverage ADCC would improve the outcome of antibody-based therapies. For instance, innate immune stimulation by TLR ligands leading to efficient activation of NK cells has been reported to improve their ADCC function^53^. Furthermore, systemic infection using different virus strains is described to induce NK cell proliferation and trafficking not only to the infected sites but also to other organs in a cytokine-dependent manner^54,55^. In this line, we demonstrated that intravenous application of TVH results in strong activation of NK cells both in NHPs and mice^22^. Specifically, PBMC analysis of TVH immunized NHPs revealed the upregulation of CD69 as well as Ki67 in NK cells, indicative of potent activation and proliferation in a relevant species for clinical studies. Likewise, infection of human PBMCs with TVH resulted in the upregulation of those markers on CD16^-^CD56^bright^ and CD16^+^CD56^dim^ human NK cells, suggesting a broad ability of our platform to activate NK cells across species.

We have previously reported that DCs play a central role for MVA-CD40L-mediated immune activation. CD40L signaling leads to activation and maturation of DCs accompanied by the expression of a wide range of proinflammatory chemokines and cytokines^21,56^. Among them IL-12p70 and IL-18 are cytokines typically induced by CD40L signaling in DCs and are potent activators of IFN-γ expression in NK cells^57,58^. Indeed, IL-12p70 and IL-18 were readily detected after TVH infection of human monocyte-derived DCs and PBMCs. IFN-γ was also produced by TVH-infected human PBMCs and in NHPs 4 and 24 h after TVH immunization. IL-12p70 is key to enhance activation of NK cells resulting in killing of tumor cells that have downregulated HLA^37^, as well as tumor cells coated by TAA targeting antibodies by ADCC^38^. In line with this, TVH infection of PBMCs enhanced NK cell activation by means of CD69 expression and enhanced NK cell cytotoxic activity against K562 target cells.

Cytolytic activity of NK cells can also be augmented by chemokines such as CCL2, CCL3, CCL4, CCL5, CCL10, and CXC3L1^59^. Interestingly, CCL2 and CCL4 were detected 4 h after each TVH immunization of NHPs, which is consistent with our previous observations in mice^21,22^. Hence, this highlights the ability of TVH to repeatedly activate NK cells after each vaccine administration.

Apart from soluble factors such as IL-12p70 and IL-18, activation of NK cells by DCs can also be promoted by direct cell–cell interaction via surface molecules. It has been reported that NK cells resemble T cells in their expression pattern of several co-stimulatory molecules such as CD28 or CD27. Blockage of CD28-CD80/86 or CD27-CD70 interaction between NK cells and DCs has been shown to reduce NK cell activation and cytotoxicity^60,61^. Interestingly, we found both costimulatory receptors CD80 and CD70 upregulated upon overnight incubation of TVH infected DCs. In conclusion, TVH is a potent activator of DCs and NK cells in NHPs and human, showing similar features as our preclinical candidate in mouse models.

Even though we were not able to measure T cell activity in the current study due to the lack of material, we gained strong evidence for the great potential of TVH to activate CD8 T cells from previous studies in mice. We could show that intravenous injection of MVA expressing a tumor antigen together with murine CD40L led to the activation and expansion of tumor-specific CD8 T cells which was shown to be crucial for therapeutic efficacy^22^. Furthermore, the great potential of MVA as a T cell activator in cancer patients was also proven in clinical trials. Intravenous but also subcutaneous treatment with MVA expressing Brachyury or HER2 led to the induction of tumor specific CD8 T cells in treated patients^20,62^. Intravenous application of a live virus expressing the immunostimulatory molecule CD40L might raise some safety concerns. MVA-BN, which is the backbone used for TVH, is a highly attenuated vaccinia virus strain that cannot replicate in human cells as its replication ability is largely restricted to embryonic avian cells^63^. MVA was approved by the FDA (JYNNEOS®) as a non-replicating vaccine against smallpox and monkeypox^1^ and its safety^64^ has been extensively proven. Despite the excellent safety profile of MVA-BN, addition of CD40L could have an impact on toxicity. Agonist anti-CD40 antibody treatment has been associated with toxicity in the clinic leading to dose limitation and, consequently, minimal clinical responses. Treatment-induced cytokine release syndrome can occur within hours and in some cases, lymphopenia and hepatotoxicity was observed that can persist for up to several weeks^64–67^. Agonist CD40 antibody-related side effects have been linked to multiple factors including CD40-induced uncontrolled activation of immune cells as well as FcyR-mediated ablation of cells. In contrast to the excessive availability of antibody in the blood after systemic administration of anti-CD40 antibodies, TVH relies on the expression of the membrane-bound natural ligand CD40L by infected cells. This ensures CD40L-dependent activation of cells in a more controlled manner and eliminates any side effects driven by antibody Fc recognition. Nevertheless, a careful dose escalation is needed in the clinic to mitigate any risks of toxicity. In addition, as for other immune therapeutics, IL-6R blocking antibodies or corticosteroids needs to be available for rapid intervention in patients with onset of first CRS symptoms.

This assumption was confirmed by the repeat-dose toxicity study in NHPs. The application of three doses of up to 6.75 × 10^9^ Inf.U of TVH administered IV in three-week intervals to NHPs as a cancer immunotherapy demonstrated an acceptable safety profile and was locally well tolerated in animals. None of the clinical observations or the findings in clinical or anatomic pathology were considered severe as many findings were reversible and none of them caused harm to the animals by impairing their physical capacity.

In summary, this study confirmed the mechanism of action previously proposed by our mouse data with MVA-CD40L. Intravenous application of TVH led to systemic activation of innate immunity ultimately resulting in enhanced NK cell activation and function. Enhanced ADCC function of NK cells can synergize with tumor targeting antibodies and thus leverage efficacy of antibody-based therapies. Our results highlight the safety and therapeutic potential of TVH. A Phase 1 open label trial has been initiated in patients with advanced Brachyury- and/or HER2-expressing cancer treated intravenously with TVH vaccine (NCT04246671).

## Materials and methods

### Ethics statement

The NHP study was carried out in accordance with the approved guidelines for animal experiments at Covance Laboratories GmbH (Münster, Germany). The experimental protocol was approved by the government (LANUV) of North Rhine-Westphalia. All experiments were performed in compliance with the ARRIVE guidelines (Animal Research: Reporting of In Vivo Experiments). The repeat-dose toxicity study was conducted in compliance with OECD Principles of good laboratory practice (GLP), as revised in 1997. Purpose-bred naïve cynomolgus monkeys (*Macaca fascicularis*) of Vietnamese origin were selected to provide 20 healthy animals of each sex. Animals of the same sex were housed in pairs or groups. Tap water was provided ad libitum. Twice daily, each pair or group of animals was offered a certified lab diet (LabDiet 5048). Animals regularly received fresh fruit and vegetables as food supplement. Animals were housed in a climate-controlled room, with a minimum of eight air changes/hour and with a cycle of 12 h light and 12 h dark.

### Cells

The BALB/3T3 clone A31, THP-1, K562, CT26.WT and HeLa cell lines were obtained from ATCC or the European Collection of Cell Cultures. The CT26 murine colon carcinoma cell line expressing human HER2 (CT26.HER2) was licensed from the Regents University of California^68^. CT26.WT, CT26.HER2 and BALB/3T3 clone A31 were cultured in DMEM Glutamax medium supplemented with 10% FCS, 1% NEAA, 1% Sodium Pyruvate and 1% Penicillin/Streptomycin (DMEM-10). HeLa cells were cultured in RPMI-1640 Glutamax medium supplemented with 10% FCS and 0.1 mg/ml Gentamicin. THP-1 cells were cultured in RPMI-1640 Glutamax medium supplemented with 10% FCS, 1% NEAA, 1% Sodium Pyruvate and 1% Penicillin/Streptomycin (RPMI-10). K562 cells were cultured in IMDM supplemented with 10% FCS, 1% NEAA, 1% Sodium Pyruvate and 1% Penicillin/Streptomycin (IMDM-10). All cell culture reagents were obtained from Gibco. All cells were grown in incubators at 37 °C and 5% CO_2_.

THP-1 cells were differentiated into macrophages in the presence of 200 ng/ml Phorbol-12-myristate-13-acetate (PMA) for 3 days followed by 2 days of rest^69^. Cells were further cultured in RPMI-10. To generate monocyte-derived DCs from healthy donors, monocytes were isolated from buffy coats according to manufacturer´s instructions using StraightFrom™ Buffy Coat CD14 MicroBead Kit (Miltenyi Biotec). Differentiation of monocytes into monocyte-derived DCs was performed using Mo-DC Differentiation Medium (Miltenyi Biotech) according to manufacturer´s instructions. Monocyte-derived DCs were further cultured in RPMI supplemented with 10% FCS, 1% Penicillin/Streptomycin and beta-mercaptoethanol (Sigma-Aldrich). Cryopreserved Peripheral Blood Mononuclear cells (PBMCs) were obtained from CTL Europe GmbH. Cryopreserved human and NHP PBMCs were thawed using CTL Anti-Aggregate Wash Solution according to manufacturer´s instructions (CTL Europe GmbH).

### Generation of MVA-BN recombinants

MVA-BN® was developed by Bavarian Nordic and is deposited at the European Collection of Cell Cultures (ECACC) (V00083008). Recombinant viruses were generated using standard methods^70^. TAEK-VAC-HerBy (TVH) encodes the full-length membrane-bound form of human CD40 ligand (hCD40L, alternatively classified as CD154) and two tumor-associated antigens (TAAs): human HER2 (hHER2, alternatively known as ERBB2) and the human transcription factor Brachyury (hBrachyury, alternatively known as T-gene).

To abrogate trastuzumab binding to TVH-encoded HER2, residues E580, F595 as well as K615 were mutated to alanine^30,31^. To abrogate pertuzumab binding to TVH-encoded HER2, residues H267, F279, V308, S310, K333, P337, L317 and H318 were mutated to alanine. To avoid any potential adverse effects upon HER2 overexpression by TVH (i) the extracellular dimerization domain was mutated, (ii) the tyrosine kinase activity was inactivated, and (iii) potential phosphorylation sites were eliminated. The introduced mutations close to the above described pertuzumab binding site will already affect heterodimer formation. To further prevent dimerization, residues D277 and E280 were mutated to arginine and lysine, respectively. Kinase activity was ablated by substituting residue K753 in the tyrosine kinase domain to methionine. To eliminate phosphorylation at known intracellular tyrosine residues amino acids 1139 to 1248 were deleted, and Y1023 was mutated to alanine.

In Brachyury, a potential nuclear localization signal (aa 286–293) was deleted to prevent localization of the protein into the nucleus and thereby inhibit its transcriptional activity. CD40 ligand encoded by TVH was left unmodified. The cDNAs of all three transgenes were codon optimized and adapted to avoid large stretches of identity or repeated sequences in or between the genes and synthesized by GeneArt (Regensburg, Germany). The HER2, CD40L, and Brachyury genes were under control of the MVA version of the vaccinia virus B2R promoter, designated as Pr1328, the MVA1-40 K promoter derived from the MVA095R gene (vaccinia virus H5R gene), and the Pr13.5long promoter^71^, respectively. All three promoters belong to the E1.1 and immediate early (IE) groups of poxviral promoters, driving very early gene expression^72,73^. Transgenes with their respective promoters were inserted into the intergenic region (IGR) 88/89 of MVA-BN. Genetically pure stocks were used for production of research grade material in chicken embryo fibroblasts, and infectious titers were determined as tissue culture infectious dose 50 (TCID_50_) per mL.

### Infection of cells

For the assessment of transgene expression, 1 × 10^6^ HeLa cells were infected with TVH using 4 Inf.U per cell overnight. To assess TVH encoded antigen loading into HLA, THP-1 derived macrophages cultured in 100 mm^2^ cell culture dishes were infected using 4 Inf.U per cell. To address TVH induced activation and maturation of DCs, monocyte-derived DCs from healthy donors were infected overnight with TVH at the indicated Inf.U. Supernatants were collected afterwards for cytokine/chemokine analysis. To address the activation of human PBMCs, cells were infected with TVH using 0.5 Inf.U per cell overnight. 3.75 µg/ml Concanavalin A (ConA, Sigma-Aldrich) was used as positive control. Supernatants were collected afterwards for cytokine/chemokine analysis. All cells were further processed for flow cytometry analysis.

### NK cell effector target killing assay

To address NK cell killing, PBMCs were infected with TVH or activated with ConA as described above. NK cells were magnetically sorted using MojoSort Human NK Cell Isolation Kit according to manufacturer´s instructions (Biolegend). K562 target cells were fluorescently labelled using Cell Proliferation Dye 670 (CPD670). Effector and target cells were co-cultured in 96 well plates at a ratio of 1:1 overnight in an incubator at 37 °C and 5% CO_2_. Next day, cells were harvested and analyzed by flow cytometry.

### NHP repeat dose toxicity study

Toxicity of TAEK-VAC-HerBy, following three intravenous bolus administrations of two different doses (Low = 1 × 10^9^ Inf.U; High = 6.75 × 10^9^ Inf.U) to cynomolgus monkeys (n = 5 males and 5 females) was assessed in 3-week intervals (vaccine administrations at Day 1, 22, 43). As control tris-buffered saline (TBS) was used. An assessment of delayed onset toxicity and/or reversibility of toxicity was made during a 4-week recovery period (study final at Day 71). Assessment of toxicity was based on clinical observations, body weights, body temperature, food consumption, assessment of feces consistency, ophthalmology, electrocardiography, and clinical and anatomic pathology evaluations. Complete necropsies were performed on all animals, with a recording of macroscopic abnormalities for all tissues. Organ weights and microscopic examinations were conducted.

Blood specimens were collected predose and on Days 2, 24, and 45, and before dosing on Days 22 and 43, as well as on Days 50 and 71 for the recovery group only for hematology, coagulation and clinical chemistry. Urine samples (after approximately 2 h without food) were collected from all animals once during the predose phase and on Days 4, 8, 25, 43, and 46 of the dosing phase and from all surviving animals during the last week of the recovery phase.

### Statistical analysis

Statistical analyses were performed as described in the figure legends using GraphPad Prism version 8.4.3 for Windows (GraphPad Software, La Jolla, CA). For immunological data, data shown are the Mean and Standard Error of the Mean (SEM). Analysis of variance (one or two-way ANOVA) with multiple comparisons tests were used to determine statistical significance between treatment groups.

## Supplementary Information

Supplementary Information 1.

Supplementary Information 2.

Supplementary Information 3.

Supplementary Information 4.

## Data Availability

The authors declare that the data supporting the findings of this study are available within the paper and its supplementary information files or available upon reasonable request from the corresponding author mahi@bavarian-nordic.com.
