# Supplementary material for: Preclinical development of a first-in-class vaccine encoding HER2, Brachyury and CD40L for antibody enhanced tumor eradication
Source: Sci Rep. 2023 Mar 30;13:5162. doi: 10.1038/s41598-023-32060-2 (PMC10060934; doi:10.1038/s41598-023-32060-2)

Supplementary Figure 1

1 MELAALCRWG LLLALLPPGA ASTQVCTGTD MKLRLPASPE THLDMLRHLY QGCQVVQGNL

61 ELTYLPTNAS LSFLQDIQEV QGYVLIAHNQ VRQVPLQRLR IVRGTQLFED NYALAVLDNG

121 DPLNNTTPVT GASPGGLREL QLRSLTEILK GGVLIQRNPQ LCYQDTILWK DIFHKNNQLA

181 LTLIDTNRSR ACHPCSPMCK GSRCWGESSE DCQSLTRTVC AGGCARCKGP LPTDCCHEQC

241 AAGCTGPKHS DCLACLHFNH SGICEL**A**CPA LVTYNT**R**T**AK** SMPNPEGRYT FGASCVTACP

301 YNYLSTD**A**G**A** CTLVCP**AA**NQ EVTAEDGTQR CE**A**CSK**A**CAR VCYGLGMEHL REVRAVTSAN

361 IQEFAGCKKI FGSLAFLPES FDGDPASNTA PLQPEQLQVF ETLEEITGYL YISAWPDSLP

421 DLSVFQNLQV IRGRILHNGA YSLTLQGLGI SWLGLRSLRE LGSGLALIHH NTHLCFVHTV

481 PWDQLFRNPH QALLHTANRP EDECVGEGLA CHQLCARGHC WGPGPTQCVN CSQFLRGQEC

541 VEECRVLQGL PREYVNARHC LPCHPECQPQ NGSVTCFGP**A** ADQCVACAHY KDPP**A**CVARC

601 PSGVKPDLSY MPIW**A**FPDEE GACQPCPINC THSCVDLDDK GCPAEQRASP LTSIISAVVG

661 ILLVVVLGVV FGILIKRRQQ KIRKYTMRRL LQETELVEPL TPSGAMPNQA QMRILKETEL

721 RKVKVLGSGA FGTVYKGIWI PDGENVKIPV AI**M**VLRENTS PKANKEILDE AYVMAGVGSP

781 YVSRLLGICL TSTVQLVTQL MPYGCLLDHV RENRGRLGSQ DLLNWCMQIA KGMSYLEDVR

841 LVHRDLAARN VLVKSPNHVK ITDFGLARLL DIDETEYHAD GGKVPIKWMA LESILRRRFT

901 HQSDVWSYGV TVWELMTFGA KPYDGIPARE IPDLLEKGER LPQPPICTID VYMIMVKCWM

961 IDSECRPRFR ELVSEFSRMA RDPQRFVVIQ NEDLGPASPL DSTFYRSLLE DDDMGDLVDA

1021 EE**A**LVPQQGF FCPDPAPGAG GMVHHRHRSS STRSGGGDLT LGLEPSEEEA PRSPLAPSEG

1081 AGSDVFDGDL GMGAAKGLQS LPTHDPSPLQ RYSEDPTVPL PSETDGYVAP LTCSPQPE~~**YV**~~

1141 ~~**NQPDVRPQPP SPREGPLPAA RPAGATLERP KTLSPGKNGV VKDVFAFGGA VENPEYLTPQ**~~

1201 ~~**GGAAPQPHPP PAFSPAFDNL YYWDQDPPER GAPPSTFKGT PTAENPEY**~~LG LDVPV

Supplementary Figure 2

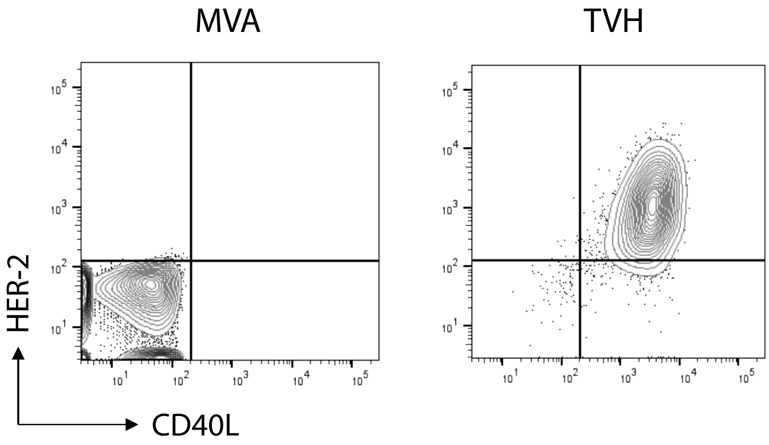

Supplementary Figure 3

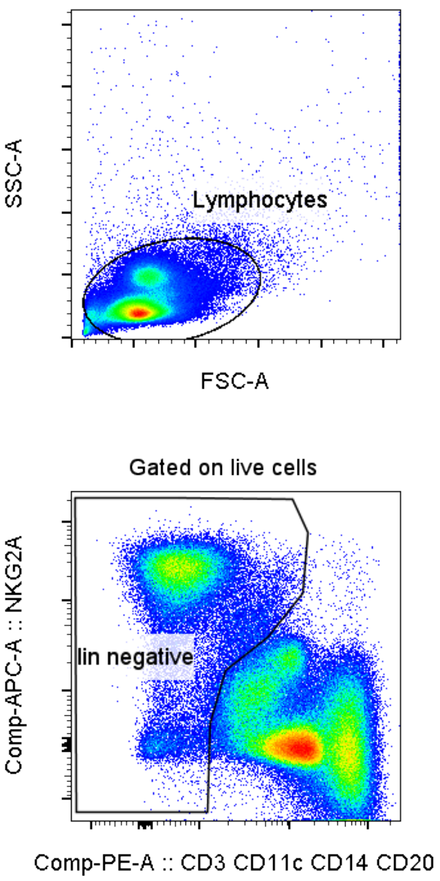

Supplementary Figure 4

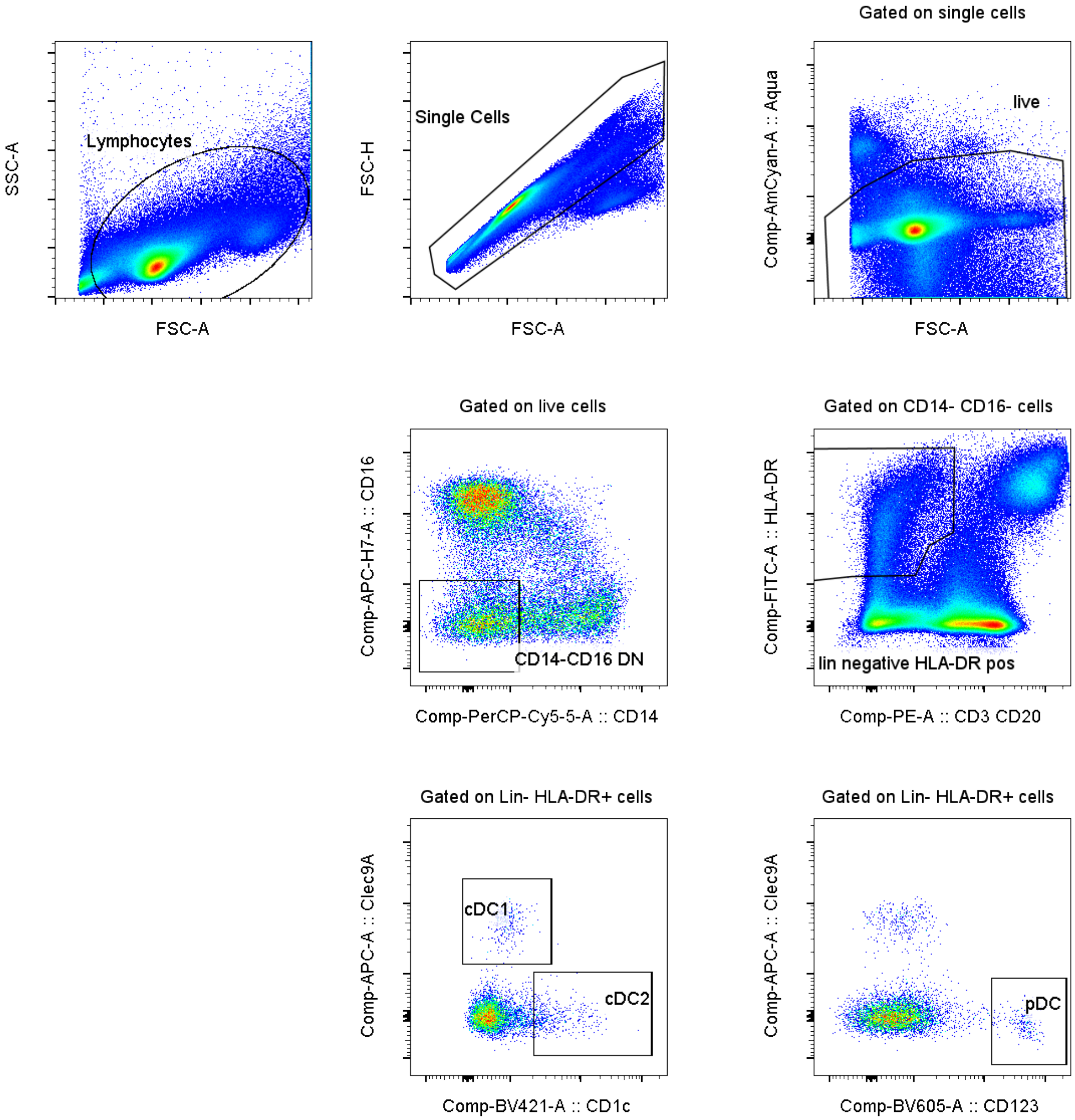

Supplementary Figure 5

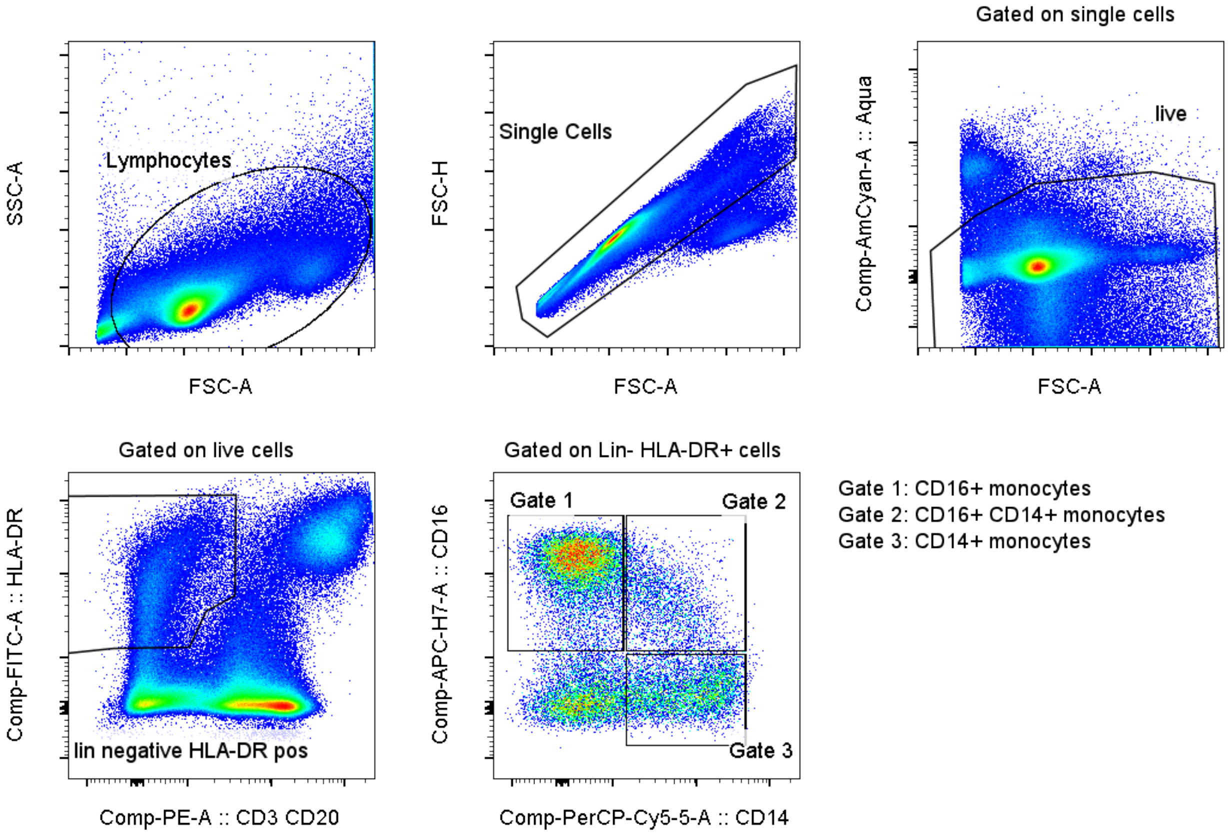

Gate 1: CD16+ monocytes
Gate 2: CD16+ CD14+ monocytes
Gate 3: CD14+ monocytes

Supplementary Figure 6

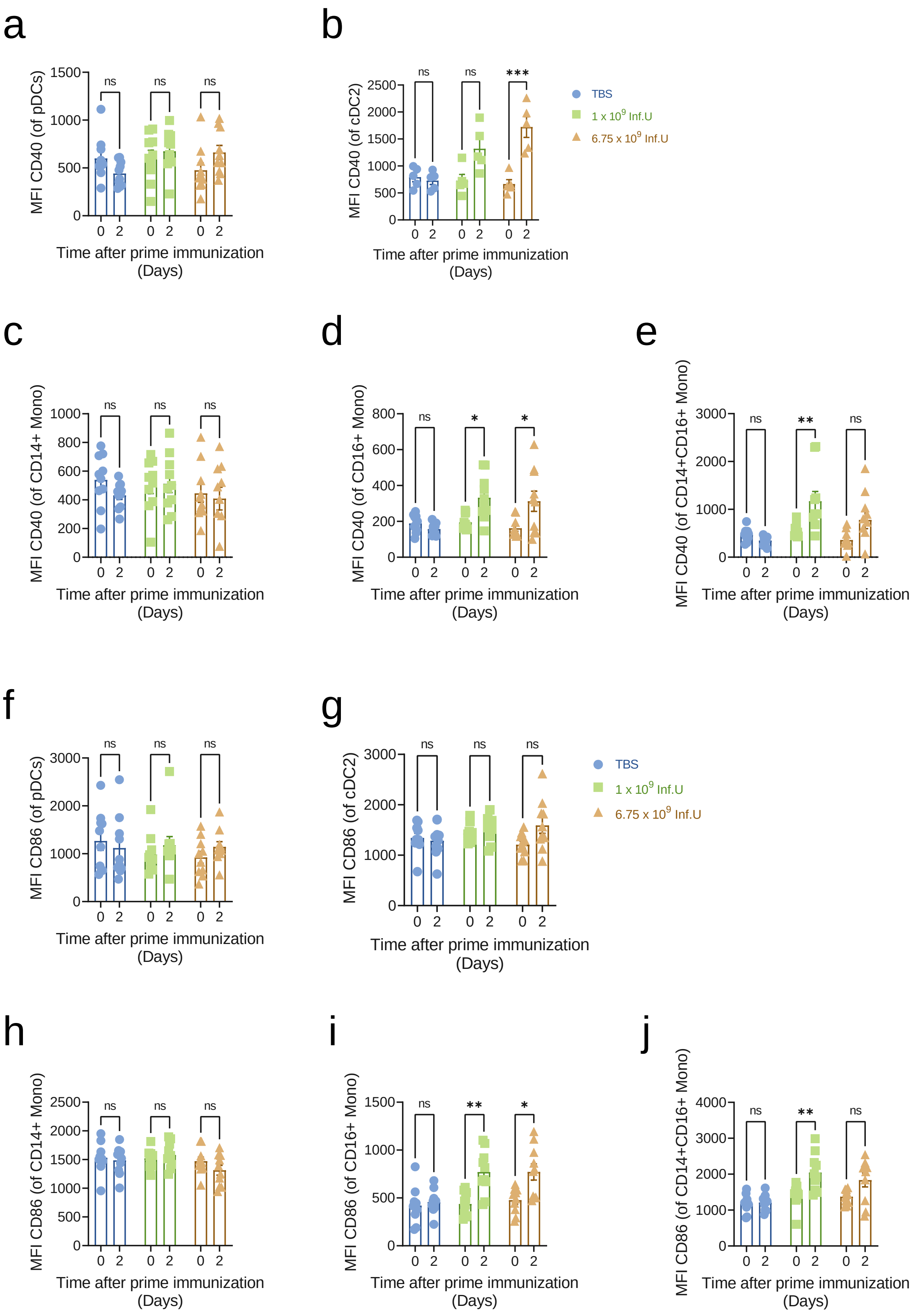

Supplement: Supplementary file 1 — Supplementary Information 1. [file 41598_2023_32060_MOESM1_ESM.pdf]
