# Supplementary material for: Preclinical development of a first-in-class vaccine encoding HER2, Brachyury and CD40L for antibody enhanced tumor eradication
Source: Sci Rep. 2023 Mar 30;13:5162. doi: 10.1038/s41598-023-32060-2 (PMC10060934; doi:10.1038/s41598-023-32060-2)
Supplement: Supplementary file 2 — Supplementary Information 2. [file 41598_2023_32060_MOESM2_ESM.pdf]

Repeated-Dose Toxicity			Test article: TVH			
Daily dose (Inf.U)	0 (control)		1×10^9		6.75×10^9	
**Sex: Number of animals**	**M: 5**	**F: 5**	**M: 5**	**F: 5**	**M: 5**	**F: 5**
**Died or sacrificed moribund**	0	0	0	0	0	0
**Body weight[(a)] [kg]**						
Predose (Day-1)	-	5.5 ± 1.61	-	4.6 ± 1.24	-	4.7 ± 1.50
Day 45	-	5.2 ± 1.45	-	4.1 ± 1.00	-	4.1 ± 1.17
**Food consumption**	-	-	-	-	-	-
**Feces observation**	-	-	-	-	-	-
Emesis of mash after dosing on Day 1	-	-	-	1	-	1
**Ophthalmoscopy**						
**Cells in aqueous humor of one or both eyes per animal**						
**Day 45**						
Grade 0.5+ (1-5 cells)	-	-	-	-	1	1
Grade 1+ (6-15 cells)	-	-	-	-	2	-
Grade 2+ (16-25 cells)	-	-	-	-	-	1
**Day 65**						
Grade 0.5+ (1-5 cells)	-	-	-	-		-
Grade 1+ (6-15 cells)	-	-	-	-	-	-
Grade 2+ (16-25 cells)	-	-	-	-	1	-
**Electrocardiography**	-	-	-	-	-	-
**Rectal Temperature [°C][(b)]**						
Predose	38.8 ± 0.27	38.6 ± 0.56	38.8 ± 0.74	38.8 ± 0.44	38.9 ± 0.38	38.2 ± 0.57
Day 1 – 6 h postdose	38.5 ± 0.73	38.5 ± 0.48	39.1 ± 0.28	39.2 ± 0.46	39.3 ± 0.13	39.1 ± 0.46
Day 22 – 6 h postdose	37.8 ± 0.25	38.7 ± 0.47	39.3 ± 0.59	39.8 ± 0.22	39.7 ± 0.40	39.4 ± 0.63
Day 43 – 6 h postdose	38.4 ± 0.36	38.2 ± 0.46	39.0 ± 0.39	39.4 ± 0.57	39.4 ± 0.11	39.5 ± 0.49
**Hematology**						
**Reticulocytes (TRET) [10E12/L][(b)]**						
Predose	0.0307 ± 0.00669	0.0890 ± 0.06511[(g)]	0.0468 ± 0.02132	0.0552 ± 0.02579	0.0600 ± 0.03593[(g)]	0.0745 ± 0.03160
Day 2	0.0381 ± 0.01080	0.0626 ± 0.00758	0.0349 ± 0.01070	0.0325 ± 0.00938	0.0351 ± 0.01799	0.0424 ± 0.00569

Repeated-Dose Toxicity			Test article: TVH			
**Daily dose (Inf.U)**	**0 (control)**		**1 x 10^9**		**6.75 x 10^9**	
**Sex: Number of animals**	**M: 5**	**F: 5**	**M: 5**	**F: 5**	**M: 5**	**F: 5**
Day 8	0.0597 ± 0.01899	0.0970 ± 0.01351	0.1035 ± 0.02976	0.1279 ± 0.02246	0.1233 ± 0.06132	0.1648 ± 0.03291
Day 22	0.0414 ± 0.01111	0.0565 ± 0.01710	0.0495 ± 0.01306	0.0690 ± 0.02493	0.0641 ± 0.03877	0.0853 ± 0.03169
**White Blood Cells (WBC) [10E9/L][(b)]**						
Predose	9.53 ± 2.246	10.60 ± 2.543[(g)]	13.33 ± 4.090	10.66 ± 3.556	10.30 ± 4.841[(g)]	11.94 ± 4.977
Day 8	13.71 ± 5.875	14.29 ± 4.473	16.77 ± 5.642	16.35 ± 3.123	27.53 ± 4.192	32.22 ± 8.980
Day 22	11.97 ± 1.907	13.82 ± 1.314	16.54 ± 4.403	14.72 ± 5.374	20.83 ± 6.500	19.71 ± 6.879
**Lymphocytes (ALYM) [10E9/L][(b)]**						
Predose	4.08 ± 1.036	4.18 ± 0.903[(g)]	4.38 ± 0.783	3.60 ± 1.507	4.59 ± 2.094[(g)]	3.91 ± 2.374
Day 2	5.44 ± 1.182	4.70 ± 1.415	3.31 ± 0.757	2.67 ± 1.089	2.56 ± 0.815	1.76 ± 1.033
Day 3	6.31 ± 1.773[(g)]	6.10 ± 1.535	4.52 ± 0.511	3.92 ± 0.766[(g)]	5.00 ± 1.549	5.01 ± 4.479
Day 8	5.85 ± 1.522	5.73 ± 1.587	10.09 ± 3.669	9.78 ± 3.359	21.09 ± 3.707	22.71 ± 6.100
Day 22	7.07 ± 1.300	6.81 ± 3.042	6.93 ± 2.003	8.50 ± 5.211	11.72 ± 2.408	11.86 ± 3.394
Day 25	4.51 ± 0.999	5.29 ± 1.771	6.40 ± 1.506	5.49 ± 1.969	8.01 ± 1.305	7.87 ± 4.814
Day 43	4.58 ± 1.450	4.03 ± 1.166	5.12 ± 0.957	4.95 ± 2.515	8.67 ± 1.806	8.09 ± 1.973
Day 46	5.66 ± 1.229	4.79 ± 0.746	5.91 ± 0.943	5.85 ± 2.475	8.15 ± 1.029	10.06 ± 3.997
Day 71	6.99 ± 0.495[(d)]	5.48 ± 2.022[(d)]	6.93 ± 0.113[(d)]	7.79 ± 3.990[(d)]	6.69 ± 2.977[(d)]	8.42 ± 2.157[(d)]
**Neutrophils (ANEU) [10E9/L][(b)]**						
Predose	4.98 ± 1.924	5.77 ± 1.910[(g)]	8.39 ± 3.466	6.46 ± 3.396	5.15 ± 2.479[(g)]	7.35 ± 2.542
Day 2	5.27 ± 1.020	7.20 ± 0.871	10.40 ± 4.711	9.73 ± 2.024	13.65 ± 1.623	13.00 ± 8.040

Repeated-Dose Toxicity			Test article: TVH			
Daily dose (Inf.U)	0 (control)		1×10^9		6.75×10^9	
Sex: Number of animals	M: 5	F: 5	M: 5	F: 5	M: 5	F: 5
Day 24	5.68 ± 2.102	6.12 ± 1.429	4.87 ± 0.979	3.31 ± 0.692	4.95 ± 1.738	3.24 ± 3.017
Day 25	6.51 ± 2.075	7.72 ± 1.123	6.25 ± 1.913	6.48 ± 6.060	4.06 ± 1.547	3.21 ± 2.884
**Basophils (ABAS) [10E9/L][(b)]**						
Predose	0.01 ± 0.009	0.02 ± 0.006[(g)]	0.01 ± 0.004	0.01 ± 0.008	0.02 ± 0.008[(g)]	0.02 ± 0.011
Day 8	0.01 ± 0.008	0.02 ± 0.011	0.09 ± 0.072	0.17 ± 0.058	0.33 ± 0.342	0.16 ± 0.244
Day 22	0.01 ± 0.011	0.02 ± 0.013	0.02 ± 0.010	0.05 ± 0.038	0.04 ± 0.036	0.04 ± 0.027
Day 24	0.02 ± 0.005	0.01 ± 0.007	0.03 ± 0.013	0.04 ± 0.016	0.12 ± 0.064	0.13 ± 0.084
Day 25	0.01 ± 0.004	0.02 ± 0.005	0.08 ± 0.033	0.08 ± 0.048	0.25 ± 0.097	0.26 ± 0.075
Day 43	0.01 ± 0.007	0.01 ± 0.005	0.02 ± 0.005	0.01 ± 0.008	0.03 ± 0.016	0.02 ± 0.012
Day 45	0.01 ± 0.000	0.02 ± 0.023	0.02 ± 0.004	0.04 ± 0.016	0.09 ± 0.043[(g)]	0.12 ± 0.038
Day 46	0.01 ± 0.015	0.02 ± 0.017	0.04 ± 0.011	0.07 ± 0.013	0.16 ± 0.080	0.18 ± 0.063
**Eosinophils (AEOS) [10E9/L][(b)]**						
Predose	0.20 ± 0.145	0.28 ± 0.162[(g)]	0.09 ± 0.054	0.21 ± 0.169	0.36 ± 0.323[(g)]	0.34 ± 0.250
Day 2	0.26 ± 0.211	0.31 ± 0.197	0.11 ± 0.073	0.19 ± 0.189	0.22 ± 0.212	0.14 ± 0.139
Day 3	0.21 ± 0.207[(g)]	0.36 ± 0.197	0.19 ± 0.102	0.05 ± 0.062[(g)]	0.21 ± 0.148	0.15 ± 0.161
Day 23	0.23 ± 0.163	0.23 ± 0.162	0.07 ± 0.039	0.10 ± 0.092	0.08 ± 0.087	0.21 ± 0.176
Day 71	0.19 ± 0.134[(d)]	0.31 ± 0.226[(d)]	0.20 ± 0.092[(d)]	0.10 ± 0.141[(d)]	0.49 ± 0.594[(d)]	0.26 ± 0.247[(d)]
**Monocytes (AMON) [10E9/L][(b)]**						
Predose	0.25 ± 0.075	0.36 ± 0.122[(g)]	0.47 ± 0.287	0.38 ± 0.293	0.19 ± 0.090[(g)]	0.32 ± 0.192

Repeated-Dose Toxicity			Test article: TVH			
**Daily dose (Inf.U)**	**0 (control)**		**1 x 10^9**		**6.75 x 10^9**	
**Sex: Number of animals**	**M: 5**	**F: 5**	**M: 5**	**F: 5**	**M: 5**	**F: 5**
Day 2	0.31 ± 0.133	0.35 ± 0.148	0.47 ± 0.141	0.27 ± 0.181	0.40 ± 0.149	0.13 ± 0.217
Day 3	0.27 ± 0.184(g)	0.48 ± 0.176	0.87 ± 0.291	0.37 ± 0.239(g)	0.76 ± 0.430	0.90 ± 1.187
Day 24	0.28 ± 0.038	0.42 ± 0.112	0.81 ± 0.434	0.61 ± 0.236	0.96 ± 0.253	1.19 ± 1.056
Day 25	0.22 ± 0.152	0.39 ± 0.078	0.65 ± 0.251	0.41 ± 0.130	0.87 ± 0.370	1.20 ± 0.351
Day 45	0.42 ± 0.282	0.51 ± 0.215	0.73 ± 0.343	0.68 ± 0.248	1.47 ± 1.427(g)	1.31 ± 0.496
Day 71	0.25 ± 0.028(d)	0.24 ± 0.085(d)	0.29 ± 0.064(d)	0.12 ± 0.054(d)	0.55 ± 0.622(d)	0.21 ± 0.007(d)
**Coagulation**						
**Platelets (PLT) [10E9/L](b)**						
Predose	381 ± 40.7	350 ± 73.5(g)	426 ± 49.7	431 ± 138.4	391 ± 92.4(g)	398 ± 52.2
Day 2	356 ± 46.1	336 ± 38.7	304 ± 41.1	272 ± 60.6	207 ± 24.8	229 ± 93.2
Day 3	344 ± 65.1(g)	331 ± 31.8	306 ± 55.9	266 ± 68.1(g)	191 ± 30.6	201 ± 111.4
Day 4	368 ± 81.8	339 ± 33.8	335 ± 72.9	270 ± 69.9	194 ± 35.9	184 ± 105.1
Day 23	342 ± 28.5	308 ± 54.6	320 ± 46.3	295 ± 70.9	232 ± 54.2	281 ± 59.5
Day 24	288 ± 78.0	302 ± 56.8	321 ± 52.3	280 ± 74.4	222 ± 47.0	257± 68.2
Day 25	349 ± 38.1	311 ± 52.5	348 ± 61.9	324 ± 76.7	235 ± 47.2	232 ± 62.6
Day 43	367 ± 41.1	318 ± 35.4	407 ± 104.9	409 ± 84.6	291 ± 90.6	403 ± 47.0
Day 44	343 ± 30.0(g)	315 ± 43.2	357 ± 94.3(g)	348 ± 72.2	209 ± 47.7(g)	300 ± 56.4
Day 45	342 ± 40.5	295 ± 34.7	379 ± 109.1	334 ± 89.0	230 ± 71.8(g)	284 ± 62.3

Repeated-Dose Toxicity			Test article: TVH			
**Daily dose (Inf.U)**	**0 (control)**		**1 x 10^9**		**6.75 x 10^9**	
**Sex: Number of animals**	**M: 5**	**F: 5**	**M: 5**	**F: 5**	**M: 5**	**F: 5**
Day 46	381 ± 60.8	327 ± 30.6	409 ± 106.4	364 ± 95.1	237 ± 58.7	298 ± 64.4
Day 71	348 ± 30.4[(d)]	287 ± 76.4[(d)]	393 ± 54.4[(d)]	354 ± 207.2[(d)]	311 ± 99.7[(d)]	390 ± 67.9[(d)]
**Activated Partial Thromboplastin Time (APTT) [s]**[(b)]						
Predose	19.9 ± 2.20	18.8 ± 1.54	19.6 ± 0.92	19.0 ± 1.76	23.2 ± 3.49	20.0 ± 1.91
Day 2	21.2 ± 2.28	20.1 ± 0.49	26.3 ± 1.67	26.3 ± 4.96	33.2 ± 4.74[(g)]	27.8 ± 4.57[(g)]
Day 3	19.6 ± 2.43	20.9 ± 1.26	21.8 ± 1.22	21.5 ± 2.24[(g)]	24.9 ± 4.08	26.9 ± 7.32
Day 4	21.5 ± 2.96	22.4 ± 0.48[(g)]	24.3 ± 6.42	23.2 ± 2.19	22.5 ± 2.33	22.8 ± 3.76
Day 8	23.8 ± 5.87[(g)]	22.7 ± 1.48[(f)]	22.4 ± 1.88	22.9 ± 2.27[(f)]	23.7 ± 3.22	22.0 ± 1.74
Day 23	20.7 ± 2.53	19.3 ± 0.66	22.1 ± 0.75	22.3 ± 3.01	26.6 ± 2.70	25.6 ± 2.49
Day 25	19.5 ± 1.97	17.7 ± 0.81	18.1 ± 0.75	17.9 ± 1.75	22.0 ± 2.03	21.8 ± 2.66
Day 44	20.2 ± 2.66	19.0 ± 1.00	20.4 ± 0.91	20.8 ± 1.74	23.9 ± 3.31	23.4 ± 2.18
**C-Reactive Protein (CRP) [mg/L]**[(b)]						
Predose	BDL	0.34 ± 0.172[(f)]	BDL	0.48 ± 0.135[(f)]	BDL	BDL
Day 2	0.49 ± 0.544[(d)]	0.34 ± 0.198[(d)]	5.11 ± 1.297	6.54 ± 0.721	6.91 ± 0.678	7.00 ± 1.626[(g)]
Day 23	0.56[(e)]	0.56 ± 0.218[(g)]	2.09 ± 1.209	4.03 ± 1.601	5.86 ± 0.933	6.08 ± 0.859
Day 24	BDL	0.36 ± 0.125[(f)]	BDL	0.73 ± 0.383[(g)]	2.28 ± 0.991	3.34 ± 1.650
Day 25	BDL	0.28 ± 0.046[(f)]	BDL	0.77[(e)]	0.74 ± 0.587[(f)]	1.01 ± 0.965[(g)]
Day 44	0.44 ± 0.516[(d)]	0.81 ± 0.537[(f)]	2.61 ± 0.620[(g)]	2.82 ± 0.417	4.30 ± 1.598	4.22 ± 0.592
Day 45	0.32 ± 0.316[(f)]	0.23 ± 0.113[(d)]	BDL	0.54 ± 0.179[(g)]	2.16 ± 2.890	1.61 ± 0.738
Day 71	0.07[(e)]	0.37[(e)]	BDL	0.07[(e)]	1.23 ± 1.506[(d)]	0.21 ± 0.177[(d)]

Repeated-Dose Toxicity			Test article: TVH			
**Daily dose (Inf.U)**	**0 (control)**		**1 x 10^9**		**6.75 x 10^9**	
**Sex: Number of animals**	**M: 5**	**F: 5**	**M: 5**	**F: 5**	**M: 5**	**F: 5**
**Immunoglobulin M (IGM) [g/L]** [(b)]						
Predose	1.22 ± 0.421	1.47 ± 0.311	1.03 ± 0.399	1.29 ± 0.342	1.26 ± 0.536	1.69 ± 0.390
Day 8	1.23 ± 0.401	1.56 ± 0.462	1.61 ± 0.432	1.95 ± 0.336	2.32 ± 0.640	2.30 ± 0.478
Day 22	1.15 ± 0.433	1.49 ± 0.478	1.35 ± 0.335	1.79 ± 0.253	2.09 ± 0.625	2.76 ± 0.446
Day 23	1.14 ± 0.421	1.41 ± 0.438	1.41± 0.378	1.78 ± 0.300	2.12 ± 0.670	2.68 ± 0.610
Day 24	1.06 ± 0.374	1.31 ± 0.380	1.29 ± 0.326	1.69 ± 0.298	1.99 ± 0.572	2.55 ± 0.479
Day 25	1.09 ± 0.347	1.38 ± 0.412	1.34 ± 0.356	1.74 ± 0.262	2.03 ± 0.597	2.51 ± 0.497
Day 43	1.08 ± 0.390	1.55 ± 0.457	1.20 ± 0.301	1.67 ± 0.277	2.17 ± 0.416	2.88 ± 0.537
Day 44	1.03 ± 0.377	1.41 ± 0.482	1.26 ± 0.389	1.62 ± 0.261	2.14 ± 0.357	2.89 ± 0.581
Day 45	1.02 ± 0.377	1.35 ± 0.404	1.24 ± 0.429	1.54 ± 0.285	1.94 ± 0.338	2.74 ± 0.492
Day 46	1.06 ± 0.354	1.46 ± 0.463	1.27 ± 0.400	1.66 ± 0.269	2.01 ± 0.253	2.82 ± 0.533
Day 71	0.90 ± 0.481[(d)]	1.53 ± 0.085[(d)]	0.77 ± 0.021[(d)]	1.62 ± 0.728[(d)]	2.36 ± 0.530[(d)]	2.69 ± 0.587[(d)]
**Urinalysis**	-	-	-	-	-	-
**Organ Weight (% difference compared with concurrent controls)**						
**Spleen**						
**Terminal Sacrifice (Day 46)**						
Absolute Weight [(c)]	6.045 g	4.264 g	41%	85%	134%	121%*
Body Weight Ratio[(c)]	0.091	0.077	54%	161%*	189%*	273%*
Brain Weight Ratio [(c)]	8.097	6.839	49%	77%	148%	99%*
**Recovery Sacrifice (Day 71)**[(d)]						
Absolute Weight [(c)]	5.152 g	4.353 g	-13%	25%	74%	133%
Brain Weight Ratio [(c)]	7.126	6.526	-11%	32%	88%	132%
**Macroscopic Observation (Day 46)**						
**Lymph Nodes**						

Repeated-Dose Toxicity			Test article: TVH			
**Daily dose (Inf.U)**	**0 (control)**		**1 x 10^9**		**6.75 x 10^9**	
**Sex: Number of animals**	**M: 5**	**F: 5**	**M: 5**	**F: 5**	**M: 5**	**F: 5**
Pancreatic lymph nodes: red discoloration and marked enlargement	0	0	0	0	1	0
Iliac lymph nodes: marked enlargement	0	0	0	0	0	0
**Spleen**						
marked enlargement	0	0	0	0	2	3
**Spleen**						
**Lymphocytes, increased cellularity, follicles**						
Slight	-	-	1	1	-	1
Moderate	-	-	-	1	1	-
Marked	-	-	2	1	2	1
**Lymphocytes, increased cellularity, red pulp**						
Minimal	-	-	-	-	-	1
Slight	-	-	1	1	-	1
Moderate	-	-	1	1	2	-
Marked	-	-	1	1	1	1
**Lymph Node, Mesenteric**						
**Lymphocytes, increased cellularity, follicles**						
Minimal	-	-	1	-	-	-
Slight	-	-	-	1	2	2
**Lymphocytes, increased cellularity, paracortical area**						
Minimal	-	-	1	-	-	-
Slight	-	-	1	1	1	3
**Lymph Node, Mandibular**						
**Lymphocytes, increased cellularity, follicles**						
Minimal	-	-	-	1	2	-
Slight	-	-	2	1	1	-
Moderate	-	-	-	-	-	-
**Lymphocytes, increased cellularity, paracortical area**						
Minimal	-	-	-	1	-	-
Slight	-	-	3	1	2	1
Moderate	-	-	-	-	1	-
**Lymph Node, Inguinal**						
**Lymphocytes, increased cellularity, follicles**						

Repeated-Dose Toxicity			Test article: TVH			
**Daily dose (Inf.U)**	**0 (control)**		**1 x 10^9**		**6.75 x 10^9**	
**Sex: Number of animals**	**M: 5**	**F: 5**	**M: 5**	**F: 5**	**M: 5**	**F: 5**
Minimal	-	-	-	1	-	-
Slight	-	-	1	2	3	1
Moderate	-	-	-	-	-	1
**Lymphocytes, increased cellularity, paracortical area**						
Slight	-	-	1	1	3	2
Moderate	-	-	2	2	-	1
**Lymph Node, Pancreatic**						
**Lymphocytes, increased cellularity, follicles**						
Marked	-	-	-	-	1	-
**Lymphocytes, increased cellularity, paracortical area**						
Marked	-	-	-	-	1	-
**Lymph Node, Iliac**						
**Lymphocytes, increased cellularity, follicles**						
Marked	-	-	-	-	-	-
**Lymphocytes, increased cellularity, paracortical area**						
Marked	-	-	-	-	-	-
**Macroscopic Observations (Terminal Sacrifice, Day 71)**[d]	-	-	-	-	-	-
**Spleen**						
**Lymphocytes, increased cellularity, follicles**						
Slight	-	-	-	-	1	-
Moderate	-	-	-	-	1	2
**Lymphocytes, increased cellularity, red pulp**						
Slight	-	-	-	-	1	1
Moderate	-	-	-	-	1	1

- No noteworthy findings; BDL = Below Detectable Limit

[a] – At necropsy; mean ± standard deviation is displayed

[b] Mean ± standard deviation is displayed

[c] Values for absolute weight and ratio of organ weights (relative to body or brain) for dosed groups expressed as percentage/X-fold control mean value (Percent control is 101%.

[d] N = 2

[e] N = 1

[f] N = 3

[g] N = 4

[#] Statistically significant difference of mean values compared for the infusion groups, Group 1 and Group 4; P<=0.05; Two-sample t-test

[*] Statistically significant difference (absolute or relative) compared with the respective control mean value; P<=0.05; ANOVA and Dunnett's
