# Supplementary material for: Preclinical development of a first-in-class vaccine encoding HER2, Brachyury and CD40L for antibody enhanced tumor eradication
Source: Sci Rep. 2023 Mar 30;13:5162. doi: 10.1038/s41598-023-32060-2 (PMC10060934; doi:10.1038/s41598-023-32060-2)
Supplement: Supplementary file 3 — Supplementary Information 3. [file 41598_2023_32060_MOESM3_ESM.docx]

**Supplementary Figure 1. Amino acid sequence of modified Her2 encoded by TAEK-VAC-HerBy (TVH; MVA-mBN484).** Mutated amino acids are shown in bold red letters and deleted amino acids as bold strikethrough letters. Shaded areas highlight the binding sites for trastuzumab (blue) and pertuzumab (yellow), the kinase domain (grey), and residues in the dimerization domain (green).

**Supplementary Figure 2. HER2 and CD40L expression in TVH infected human DCs**

Monocyte-derived DCs were generated after enrichment of CD14^+^ monocytes from human PBMCs and cultured for 7 days in the presence of GM-CSF and IL-4 explained in *Materials and Methods*. Mo-DCs were infected overnight either with MVA-BN or TVH at a MOI of 5. Next day, cells were stained and analysed by flow cytometry. Expression of HER2 and CD40L is shown on CD1a^+^ DCs.

**Supplementary Figure 3. Gating scheme of NHP NK cells**

NHP received intravenous injections of either TBS, 1x10^9^ Inf.U TVH or 6.75x10^9^ Inf.U TVH. Blood was collected 48 hours after immunization and a baseline bleeding before immunization was performed in all individuals. Analysis of NK cells was done using PBMCs, isolated from blood taken at these time points (0h, Day 0 and after 48h, Day 2). NHP PBMC were stained with L/D Aqua, NKG2A-APC, CD3-PE, CD11c-PE, CD14-PE, CD20-PE, CD8a-PerCP-Cy5.5, and analysed by flow cytometry. NK cells were identified as outlined in the gating strategy.

**Supplementary Figure 4. Gating strategy of NHP DCs**

Dendritic cell analysis was done using PBMCs, isolated from blood taken before (0h, Day 0) and after (48h, Day 2) the first TVH administration, as explained above. NHP PBMC were stained with L/D Aqua, CD16-APC-H7, CD14-PerCP-Cy5.5, HLA-DR-FITC, CD3-PE, CD20-PE, Clec9A-APC, CD1c-BV421, CD123-BV605, and analysed by flow cytometry. cDC1-DCs, cDC2-DCs and pDCs were identified as depicted in the gating strategy.

**Supplementary Figure 5. Gating strategy of NHP monocyte subsets**

Analysis of monocytes was done using PBMCs, isolated from blood taken before (0h, Day 0) and after (48h, Day 2) the first TVH administration, as explained above. NHP PBMC were stained with L/D Aqua, CD16-APC-H7, CD14-PerCP-Cy5.5, HLA-DR-FITC, CD3-PE, CD20-PE, and analysed by flow cytometry. Different monocyte subsets were identified as shown in the gating strategy.

**Supplementary Figure 6. Activation of NHP DCs and monocytes via intravenous immunization of TVH**

**(a-e)** Expression of co-stimulatory markers, CD40 and **(f-j)** CD86 on the surface of NHP DCs and monocytes. NHP received intravenous injections of either TBS, 1x10^9^ Inf.U TVH or 6.75x10^9^ Inf.U TVH. Blood was collected 48 hours after first immunization and a baseline bleeding before immunization was performed in all individuals. Activation of innate immune cells was investigated at these time points (day 0 and day 2) by checking the expression of co-stimulatory markers CD40 and CD86 by flow cytometry. **(a)** Mean Fluorescence Intensity (MFI) of CD40 on CD123^+^ pDCs, **(b)** CD1c^+^ cDC2-DCs, **(c)** CD14^+^ monocytes, **(d)** CD16^+^ monocytes and **(e)** CD16^+^ CD14^+^ monocytes; **(f)** MFI of CD86 on CD123^+^ pDCs, **(g)** CD1c^+^ cDC2-DCs, **(h)** CD14^+^ monocytes, **(i)** CD16^+^ monocytes and **(j)** CD16^+^ CD14^+^ monocytes are indicated. Data is shown as Mean±SEM. Two-way ANOVA was performed for statistical analysis. ns, non-significant; *, *p* < 0.05; **, *p* < 0.01; ***, *p* < 0.005.

**Supplementary Table 1. Clinical observations of NHP after intravenous TVH administration**

Summary of treatment related clinical observations, body weights, body temperature, food consumption, feces observations, ophthalmic examinations, electrocardiography, and clinical and anatomic pathology in the repeat-dose toxicity study conducted in NHP.
