# Supplementary material for: Preclinical development of a first-in-class vaccine encoding HER2, Brachyury and CD40L for antibody enhanced tumor eradication
Source: Sci Rep. 2023 Mar 30;13:5162. doi: 10.1038/s41598-023-32060-2 (PMC10060934; doi:10.1038/s41598-023-32060-2)
Supplement: Supplementary file 4 — Supplementary Information 4. [file 41598_2023_32060_MOESM4_ESM.docx]

**Supplementary Material and Methods**

Immunofluorescence

For immunofluorescence analysis, subconfluent BALB/3T3 clone A31 cells in 24-well plates were either mock infected or infected at a MOI of 2 with MVA recombinant TAEK-VAC-HerBy (TVH), encoding Brachyury-ΔNLS, or MVA encoding Brachyury (MVA-Brachyury), or MVA-BN. After 17 h of infection, cells were washed twice with PBS and fixed with ice-cold 100% methanol for 10 min on ice. Subsequently, cells were washed with 1x PBS and permeabilized using 1x PBS with 1% BSA, 0.5% Triton X-100, 0.2 µg/ml EDTA for 15 min on ice. Cells were rinsed and then blocked for 10 min at room temperature (RT) with incubation buffer (1x PBS with 0.5% BSA). Cells were stained using primary rabbit anti-Brachyury antibody (QC2077,1:500) for 1 h at RT and secondary PE-conjugated anti-rabbit-IgG (H+L) antibody (Jackson Immuno 711-116-152; 1:500) for 30-45 min at RT. After being washed, cells were analyzed under a fluorescence microscope. Fluorescence images were taken using a Zeiss Axio Vert.A1 microscope equipped with AxioCam ICm 1 digital camera and the ZEN 2.3 software. Note that expression of Brachyury-ΔNLS from TVH and Brachyury from MVA-Brachyury, are under control of different promoter, Pr13.5long and PrS, respectively. The MVA-Brachyury control for nuclear localization of Brachyury also encodes costimulatory molecules B7-1, ICAM-1, and LFA-3 (TRICOM).

Flow cytometry

Mononuclear cell suspensions, DCs or tumor cells were stained for 30 minutes at 4°C in the dark using fixable live/dead viability kits prior to staining (Life Technologies). Cells were stained with antibodies listed below. For Ki67 staining cells were fixed using FoxP3 Staining Kit (eBioscience). All cells were acquired using a digital flow cytometer (LSR II, BD Biosciences), and data were analyzed with FlowJo software version 10.3 (Tree Star).

List of Antibodies:

| **Antibody (Clone)** | **Fluorochrom** | **Company** |
| --- | --- | --- |
| HLA-DR (L243) | FITC | BioLegend |
| CD3(10D12) | PE | Miltenyi |
| CD20(2H7) | PE | BD |
| CD14 (M5E2) | PerCP-Cy5.5 | BioLegend |
| CD80 (L307.4) | PE-Cy7 | BD |
| Clec9A (34A/Clec9A) | APC | BD |
| CD16 (3G8) | APC780 | BioLegend |
| CD1c (L161) | BV421 | BioLegend |
| l/d | BV506 | Life Technologies |
| CD123 (7G3) | BV605 | BD |
| CD86 (IT2.2) | BV650 | BioLegend |
| CD40 (5C3) | BV785 | BioLegend |
| CD11c (3.9) | PE | BioLegend |
| CD8a (RPA-T8) | PerCP-Cy5.5 | BioLegend |
| CD137 (4B4-1) | PE-Cy7 | BioLegend |
| NKG2A (REA110) | APC | Miltenyi |
| NKG2D (1D11) | APC780 | BioLegend |
| CD69 (REA824) | BV421 | Miltenyi |
| CD56 (5.1H11) | BV605 | BioLegend |
| CD25 (M-A251) | BV785 | BioLegend |
| HLA ABC (W6/32) | BV605 | Biolegend |
| CD11b (ICRF44) | APC | BioLegend |
| CD70 (113-16) | APC | BioLegend |
| HER2 (24D2) | PE/APC | BioLegend |
| Trastuzumab |  | Invivogen |
| Pertuzumab |  | Creative-Biolabs |
| CD40L (24-31) | APC | BioLegend |
| Brachyury (rabbit) |  | Dr. Jeffrey Schlom, National Cancer Institute |
| Vaccinia (rabbit polyclonal) |  | quartett GmbH |
| hIgG Fc (M1310G05) | FITC | BioLegend |

Cytokine detection

Cytokine concentrations in cell culture supernatants were determined by Multiplex Luminex assays according to manufacturer´s instructions (Thermo Fisher). Analysis was performed using Masterplex 2010 version 2.0.0.77 (Hitachi Solutions, Ltd.).

Cytokine and chemokine induction in serum samples of NHP prior to each immunization, 4 h and 24 h after each immunization were analyzed using a Luminex 200 Analyzer. For the analysis of the analytes Milliplex MAP kits NHP (Merck Millipore) and ProcartaPlex-5-plex kits (Thermo Fisher) were used according to manufacturer´s instructions. Analysis was performed using the Luminex xPONENT 3.1 software.

Enzyme-linked immunosorbent assay (ELISA)

MVA- and HER2- specific IgG in serum of NHP was measured by direct ELISA in NHP serum samples at predose, before each booster administration (Day 22, Day 43) and at the end of the observation periods (Day 46 for all animals, Day 71 for Recovery Group). Briefly, 96-well ELISA plates were coated overnight with either MVA antigen (produced by BN) or human HER2 protein (Sino Biological).

For the detection of MVA-specific antibodies serum samples were titrated using twofold serial dilutions starting at 1:100. For the detection of HER2-specific antibodies the standard anti-HER2/neu (human IgG1; BIOZOL) was titrated using two-fold serial dilutions starting at 2 μg/mL and test samples were used at a dilution of 1:100. Rabbit anti-monkey IgG peroxidase (Sigma) was used in both ELISA as detection antibody, and all antibody titers were calculated by 4-parameter fit (Magellan Software). For MVA ELISA the average of the OD values of the negative control (naïve sample) needed to be below an OD of 0.3 and was defined as assay cut-off. Serum samples with an OD value below the assay cut-offs were negative and given the arbitrary value of 1. For HER2 ELISA, serum samples with an OD value ≤ 0.471 were reported negative and given an arbitrary value of 1700 Elisa Units, which corresponds to half of the lower limit of quantitation.

HLA ligandome analysis

Identification of TVH-derived peptides presented by PMA-induced THP-1 macrophages following infection and processing was performed by ProImmune (Oxford, UK) using the ProPresent® antigen presentation assay^1^. Briefly, THP-1 cells were differentiated into macrophages using 200ng/ml PMA, followed by a resting phase. On day 5 of differentiation, cells were infected with TVH at Inf.U 4 for 14 hours. Afterwards, infected THP-1 macrophages were harvested and washed with cold PBS prior to lysis in a detergent‐containing buffer solution. PanHLA molecules were recovered in a specific immunoaffinity step. Peptides were then eluted from the HLA complexes and processed for further analysis by sequencing mass spectrometry. Peptide samples were subsequently analyzed by high‐resolution sequencing mass spectrometry (LC‐MS/MS). Resulting data were then compiled and analyzed using sequence analysis software referencing the Human Uniprot_Complete_Proteome Database with the incorporated test item sequence. The likelihood of peptides to be real identities is described by their Expect Value and by the False Discovery Rate (FDR). The Expect Value is a statistical value describing the reliability of a given peptide. Peptides with an Expect Value ≤ 0.05 lie within a confidence range of >95% and are indicative of identity.

1 Gouw, J. W. *et al.* Identification of peptides with tolerogenic potential in a hydrolysed whey-based infant formula. *Clin Exp Allergy* **48**, 1345-1353, doi:10.1111/cea.13223 (2018).
